# The human ion channel TRPM2 modulates migration and invasion in neuroblastoma through regulation of integrin expression

**DOI:** 10.1038/s41598-022-25138-w

**Published:** 2022-11-29

**Authors:** Lei Bao, Fernanda Festa, Iwona Hirschler-Laszkiewicz, Kerry Keefer, Hong-Gang Wang, Joseph Y. Cheung, Barbara A. Miller

**Affiliations:** 1 Departments of Pediatrics, The Pennsylvania State University College of Medicine, P.O. Box 850, Hershey, PA 17033 USA; 2 Departments of Biochemistry and Molecular Biology, The Pennsylvania State University College of Medicine, P.O. Box 850, Hershey, PA 17033 USA; 3 Departments of Pharmacology, The Pennsylvania State University College of Medicine, P.O. Box 850, Hershey, PA 17033 USA; 4 Renal Medicine, Brigham and Women’s Hospital, Boston, MA 02115 USA

**Keywords:** Cancer, Cell biology, Oncology

## Abstract

Transient receptor potential channel TRPM2 is highly expressed in many cancers and involved in regulation of key physiological processes including mitochondrial function, bioenergetics, and oxidative stress. In Stage 4 non-MYCN amplified neuroblastoma patients, high TRPM2 expression is associated with worse outcome. Here, neuroblastoma cells with high TRPM2 expression demonstrated increased migration and invasion capability. RNA sequencing, RT-qPCR, and Western blotting demonstrated that the mechanism involved significantly greater expression of integrins α1, αv, β1, and β5 in cells with high TRPM2 expression. Transcription factors HIF-1α, E2F1, and FOXM1, which bind promoter/enhancer regions of these integrins, were increased in cells with high TRPM2 expression. Subcellular fractionation confirmed high levels of α1, αv, and β1 membrane localization and co-immunoprecipitation confirmed the presence of α1β1, αvβ1, and αvβ5 complexes. Inhibitors of α1β1, αvβ1, and αvβ5 complexes significantly reduced migration and invasion in cells highly expressing TRPM2, confirming their functional role. Increased pAkt^Ser473^ and pERK^Thr202/Tyr204^, which promote migration through mechanisms including integrin activation, were found in cells highly expressing TRPM2. TRPM2 promotes migration and invasion in neuroblastoma cells with high TRPM2 expression through modulation of integrins together with enhancing cell survival, negatively affecting patient outcome and providing rationale for TRPM2 inhibition in anti-neoplastic therapy.

## Introduction

Transient receptor potential (TRP) channels are members of a superfamily of cation-permeable ion channels involved in many physiological processes. The TRPM (melastatin) subfamily has many members involved in proliferation and cell survival^1–4^. TRPM2, the second member of this subfamily to be cloned, is a cation channel widely expressed in many cell types. TRPM2 is activated by processes in vivo which increase ADP-ribose (ADPR) production, including oxidative stress, which occurs in tumor cells; ADPR then binds to sites in the TRPM2 C-terminus (NUDT9-H domain) and N-terminus to open the channel by effecting conformational changes^5–8^. TRPM2 is positively regulated by intracellular calcium^9,10^ and inhibited by acidification^11^. TRPM2 is highly expressed in a number of cancers including breast, lung, pancreas, prostate, neuroblastoma, and leukemia^2,12–14^. The increased expression of TRPM2 in malignant cells is consistent with its demonstrated role in promoting cancer cell proliferation and survival^2,14–19^.

Neuroblastoma is the most common extracranial solid tumor of childhood. While early stage disease is highly curable, high risk patients who present with metastatic disease have a long term survival rate of less than 50%^20^. TRPM2 is highly expressed at both the RNA and protein level in many neuroblastomas^13,21^. When TRPM2 is inhibited by a dominant splice variant^13^ or deleted using CRISPR technology^19^, neuroblastoma cell proliferation is reduced in vitro and in xenografts, and sensitivity to doxorubicin is significantly increased. TRPM2 regulates cell cycle progression through modulation of expression of transcription factors E2F1 and FOXM1^22^. TRPM2 supports neuroblastoma cell survival through maintenance of mitochondrial function, sustaining expression of mitochondrial electron transport chain proteins and the mitochondrial calcium uniporter (MCU), mediated via CREB and HIF-1α expression, and MCU activation through a calcium-dependent mechanism^13,14,19,23^. Mitochondrial membrane potential, oxygen consumption rate, calcium entry through the MCU, and bioenergetics including ATP production are maintained when TRPM2 is expressed and are significantly decreased in TRPM2 inhibition or deletion. In addition, TRPM2 is important in reducing oxidative stress. In TRPM2 inhibition or deletion, production of reactive oxygen species (ROS) is significantly increased through dysfunctional mitochondria^14,19^. The antioxidant response is impaired in cells with TRPM2 deletion through decreased expression of Nrf2, a transcription factor which regulates many antioxidant enzymes and cofactors GSH, NADPH, and NADH^24^, leading to high ROS levels and decreased cell viability. In neuroblastoma, calcium entry through TRPM2 is also important for maintaining DNA repair, and in TRPM2 deletion, increased ROS results in increased DNA damage, further exacerbated by reduced expression of DNA repair proteins^22^. This is partially mediated through TRPM2 modulation of expression of the master transcriptional regulators E2F1 and FOXM1, which function in cell proliferation, cell cycle progression, DNA repair, migration, and metastasis^22,25–30^. Together, the roles of calcium entry thorough TRPM2 in modulating mitochondrial function, bioenergetics, ROS levels, and DNA repair combine to sustain neuroblastoma proliferation and viability, which are significantly impaired in TRPM2 blockade or deletion. These data suggested that inhibition of TRPM2 maybe a novel therapeutic approach to reduce proliferation and survival of neuroblastoma and other malignancies in which TRPM2 is highly expressed.

Calcium signaling contributes to metastasis through complex and interacting pathways and mechanisms including involvement of epithelial-mesenchymal transition (EMT), cell migration and invasion, and angiogenesis^31–33^. TRPM2 itself has recently been shown to promote migration and invasion of gastric^34^ and pancreatic cancer^35^. In pancreatic cancer, high TRPM2 expression was associated with tumor proliferation, invasive ability, and poor prognosis, although the mechanism was not defined. In gastric cancer, TRPM2 downregulation inhibited migration and invasion and was associated with decreased EMT markers, integrins, Akt phosphorylation and increased PTEN. Integrins are key integral membrane proteins which anchor cells to different substrates in the extracellular matrix by linking to actin filaments through intracellular mediator proteins. Among the integrins, integrin β1 has a primary role in migration and metastasis of melanoma^36^, pancreatic cancer^37^, and breast cancer^38^. A number of the transcription factors which regulate integrin expression are reduced in TRPM2 inhibition or depletion including HIF-1^19,39^, E2F1^22^, and FOXM1^22,28^, suggesting that TRPM2 may regulate migration and invasion of neuroblastoma cells through transcriptional modulation of integrin expression.

Here, the role of TRPM2 in migration and invasion of neuroblastoma cells was examined. Major findings are: (1) Stage 4 non-MYCN amplified neuroblastoma patients with high TRPM2 expression have significantly worse outcome; (2) high TRPM2 expression significantly increases migration and invasion; (3) in cells highly expressing TRPM2, RNA sequencing analysis, RT-qPCR, and Western blotting demonstrated increased expression of α1, αv, β1, and β5 integrins, with the majority of α1, β1, and αv integrins localized in the membrane fraction; (4) co-immunoprecipitation demonstrated α1β1, αvβ5, αvβ1 integrin complexes and antagonists of α1β1, αvβ5, and αvβ1 activity reduced migration and invasion of cells with high TRPM2 expression; (5) in cells with increased TRPM2, high expression of HIF-1α, E2F1, and FOXM1 transcription factors, which are modulated by TRPM2 and bind to α1, αv, β1 and β5 integrin promoters/enhancers, was associated with greater integrin expression and increased migration and invasion; and (6) increased activation of Akt and ERK in cells with high TRPM2 expression contributed to increased migration and invasion. These data demonstrate that TRPM2 has key roles in regulating the metastatic potential of neuroblastoma cells.

## Results

### TRPM2 is highly expressed in many cancers including neuroblastoma

**Figure 1:**
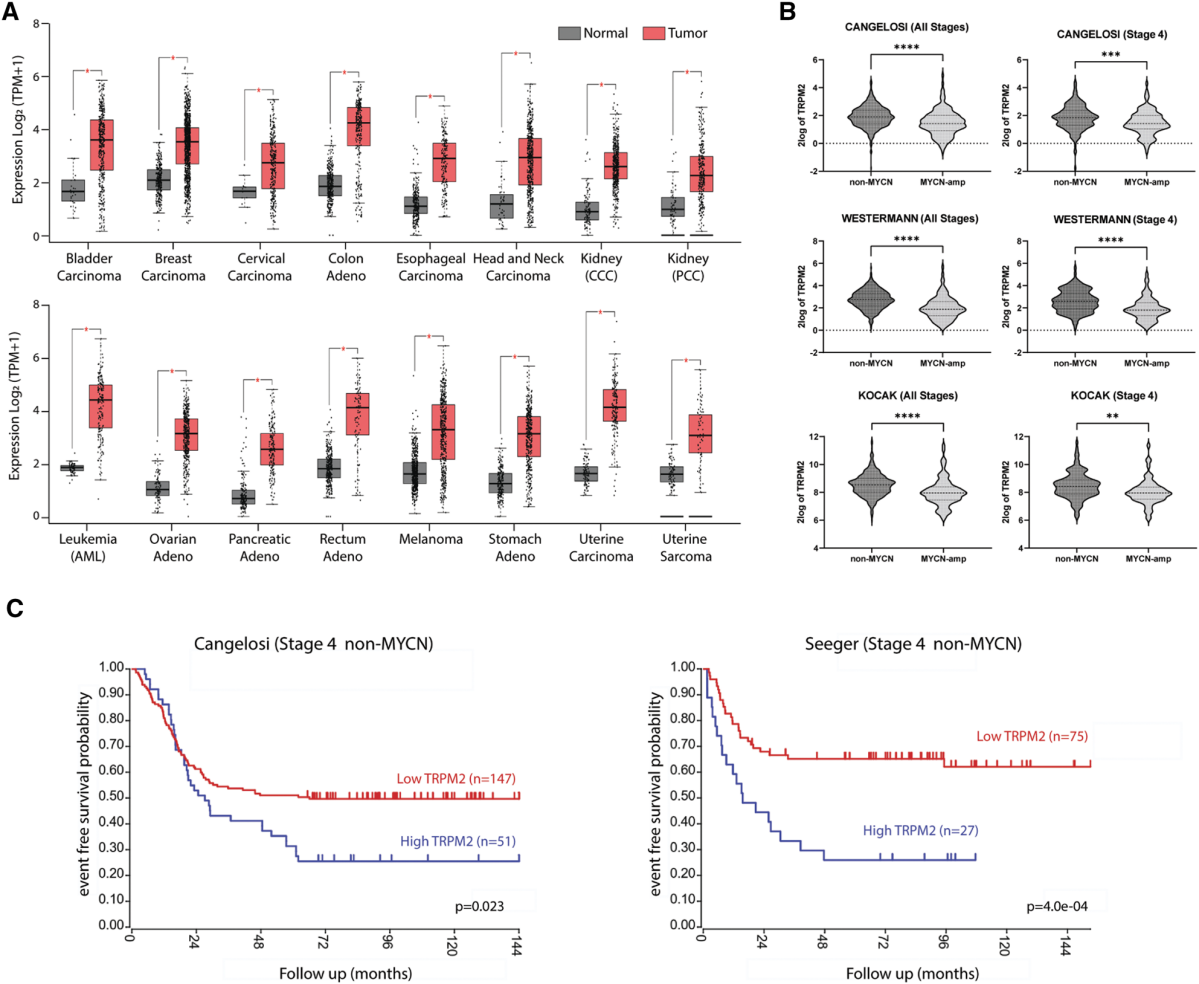
TRPM2 is highly expressed in many cancers. (**A**) TRPM2 expression is increased in many malignancies compared to normal tissue. The GEPIA2 tool was used to analyze and compare TCGA tumor datasets to TCGA and GTEx normal datasets (See "Materials and Methods"). The number of samples for each tumor type are shown in Supplementary Table 1. The median 25–75 percentiles are boxed and the 10–90 percentiles for each group shown with lines. The median is shown with a bar. Significant differences were assessed with one-way ANOVA. *p < 0.01. (**B**) Expression of TRPM2 was analyzed in neuroblastoma samples from all stages of disease and in Stage 4 using three databases in the R2 platform (Cangelosi^40^, Westermann, Kocak^41^ databases; Supplementary Table 2). Expression levels of TRPM2 were compared between samples with and without MYCN amplification across all stages of disease (left panel) or in Stage 4 (right panel). Data was analyzed by unpaired t-test (**p < 0.01, ***p < 0.001, ****p < 0.0001). (**C**) Kaplan Meier survival plot for Stage 4 neuroblastoma patients without MYCN amplification divided based on level of TRPM2 expression in tumors. Samples in the last quartile for TRPM2 expression were designated High TRPM2 (blue), while remaining samples were grouped into Low TRPM2 (red). Two independent datasets with survival data were used: Cangelosi database^40^ (n = 198, *p* < 0.023); Seeger database^42^ (n = 102, *p* < 0.00029); analyzed with one-way ANOVA.

TRPM2 is highly expressed in many malignancies compared to normal tissues (Fig. 1A, Supplementary Table 1; TCGA, GTEx databases). High expression of TRPM2 in neuroblastoma was demonstrated previously^13,21^. Here, expression of TRPM2 was determined to be significantly higher in non-MYCN amplified neuroblastoma tumors compared to MYCN amplified tumors at all stages and specifically in Stage 4 disease, using three databases from the R2 platform (R2:Genomics Analysis and Visualization Platform; http://r2.amc.nl), chosen for analysis because of their high number of neuroblastoma samples (Fig. 1B, Supplementary Table 2)^40,41^. In Stage 4 neuroblastoma patients without MYCN amplification, high TRPM2 expression correlated with worse patient event free survival compared to patients with low TRPM2 levels (Fig. 1C)^40,42^, suggesting that in this subgroup TRPM2 has an important role in modulating metastatic and/or refractory disease. In Stage 4 MYCN amplified neuroblastoma patients, TRPM2 expression did not correlate significantly with outcome, possibly because MYCN itself transcriptionally regulates expression of many oncogenic proteins including FOXM1^43^.

### High TRPM2 levels increase migration and invasion in neuroblastoma

**Figure 2:**
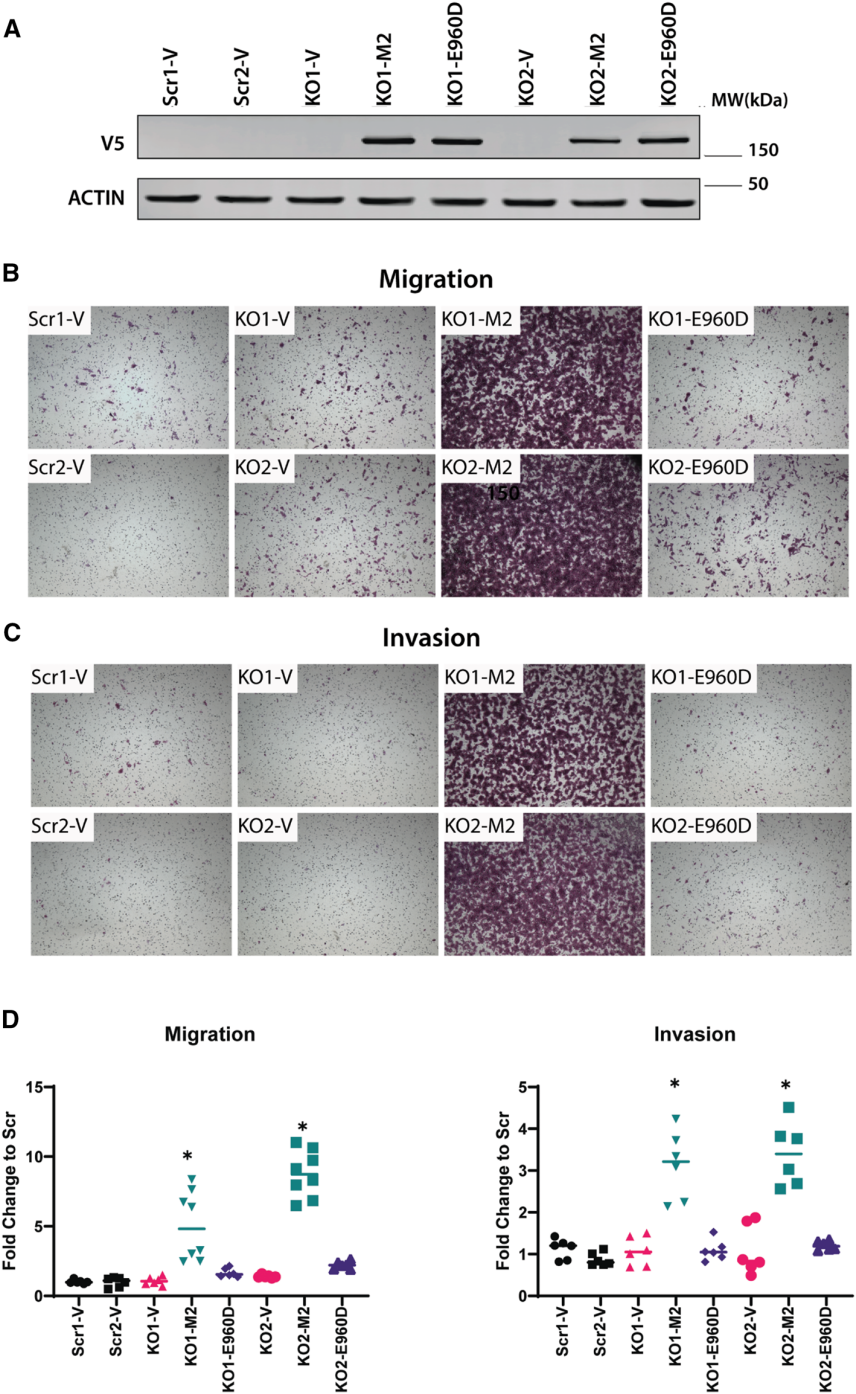
High TRPM2 expression increases invasion and migration in neuroblastoma. (**A**) Western blotting was performed on two clones of SH-SY5Y scrambled control cells (Scr1-V, Scr2-V), TRPM2 KO (KO1-V, KO2-V), and TRPM2 KO cells reconstituted with either TRPM2 (KO1-M2, KO2-M2) or the E960D calcium-impermeant TRPM2 mutant (KO1-E960D, KO2-E960D). Probing with anti-V5 antibody demonstrated successful TRPM2 transfection. Full length gels for Western blots are shown in Supplementary Fig. S1. Migration (**B**) and invasion (**C**) assays were performed as described in Methods, and representative pictures from Boyden chambers are shown. (**D**) Analysis of migration and invasion density of two clones each of scrambled, KO, and KO cells reconstituted with TRPM2 or E960D was performed. Symbols indicate individual wells and are shown for Scr-V (black), KO-V (red), KO-M2 (green), and KO-E960D (blue) cells. Medians are indicated for 3 independent experiments with a line. Each of the three experiments had 2 wells/clone/group (total replicates = 6) except for the KO-M2 clones in one migration experiment, which had 4 wells/group (replicates = 8). Statistical difference of each group compared to scrambled controls were analyzed by one-way ANOVA. *p < 0.0.0001.

The role of TRPM2 in migration and invasion of neuroblastoma was studied further using SH-SY5Y cells depleted of TRPM2 with CRISPR technology (KO1-V, KO2-V), TRPM2 KO cells reconstituted with full length TRPM2 (KO1-M2, KO2-M2) or the TRPM2 Ca^2+-^impermeant mutant E960D (KO1-E960D, KO2-E960D), or scrambled control cells generated at the time of CRISPR KO (Scr1-V, Scr2-V) (Fig. 2)^19^. Characterization of TRPM2 deletion in these cells was reported previously and TRPM2 knockout documented by RT-PCR and Western blotting^19^. The level of TRPM2 in KO cells reconstituted with wild type TRPM2 was similar to that in cells reconstituted with the TRPM2 calcium impermeant mutant E960D (Fig. 2A), but higher than levels in scrambled control cells. Migration and invasion were examined with Boyden chambers. KO cells reconstituted with TRPM2 had significantly greater migration and invasion than scrambled control or KO cells (Fig. 2B–D), which were similar to each other. Cells stably transfected with the TRPM2 Ca^2+-^impermeable mutant E960D showed migration and invasion ability similar to scrambled control and KO cells. The E960D mutant differs from wild type TRPM2 in one amino acid in the pore region, and loss of calcium gating was demonstrated in our laboratory^19,44,45^. This demonstrates that calcium influx through TRPM2 has a role in the increased migration/invasion seen in cells highly expressing TRPM2.

### Integrin expression is increased in neuroblastoma cells with high TRPM2 expression

To determine the mechanisms through which increased TRPM2 enhances migration and invasion, RNA sequencing (seq) analysis was performed. Our analysis compared mRNA levels in SH-SY5Y neuroblastoma cells with TRPM2 deletion (KO) to the same cells stably transfected with TRPM2. Top cell signaling pathways identified as different in cells with high TRPM2 expression are shown in Supplementary Fig. S2. These included RNAs involved in “HIF-1α Signaling”, “PI3K/AKT Signaling”, “ERK/MAPK Signaling”, and “CREB Signaling in Neurons”. These pathways were selected for further study because they previously were observed to be regulated by TRPM2 and they have roles in migration and invasion. RNA seq data discussed in this manuscript are deposited in NCBI’s Gene Expression Omnibus^46,47^ and are accessible through GEO Series accession number GSE203660. Analysis of the “HIF-1α Signaling” demonstrated a significant increase (greater than twofold) in HIF-1α mRNA in cells stably transfected with TRPM2, confirming previous studies that HIF-1α expression is modulated by TRPM2^13,19^. The increase in expression of mRNAs in the “PI3K/AKT Signaling” pathway and “ERK/MAPK Signaling” pathway was driven by increases in members including integrins; however, increases in Akt or ERK1/2 mRNA themselves were not found. “CREB Signaling in Neurons” included increased CREB3 Regulatory Factor (CREBRF) but not CREB itself.

**Figure 3:**
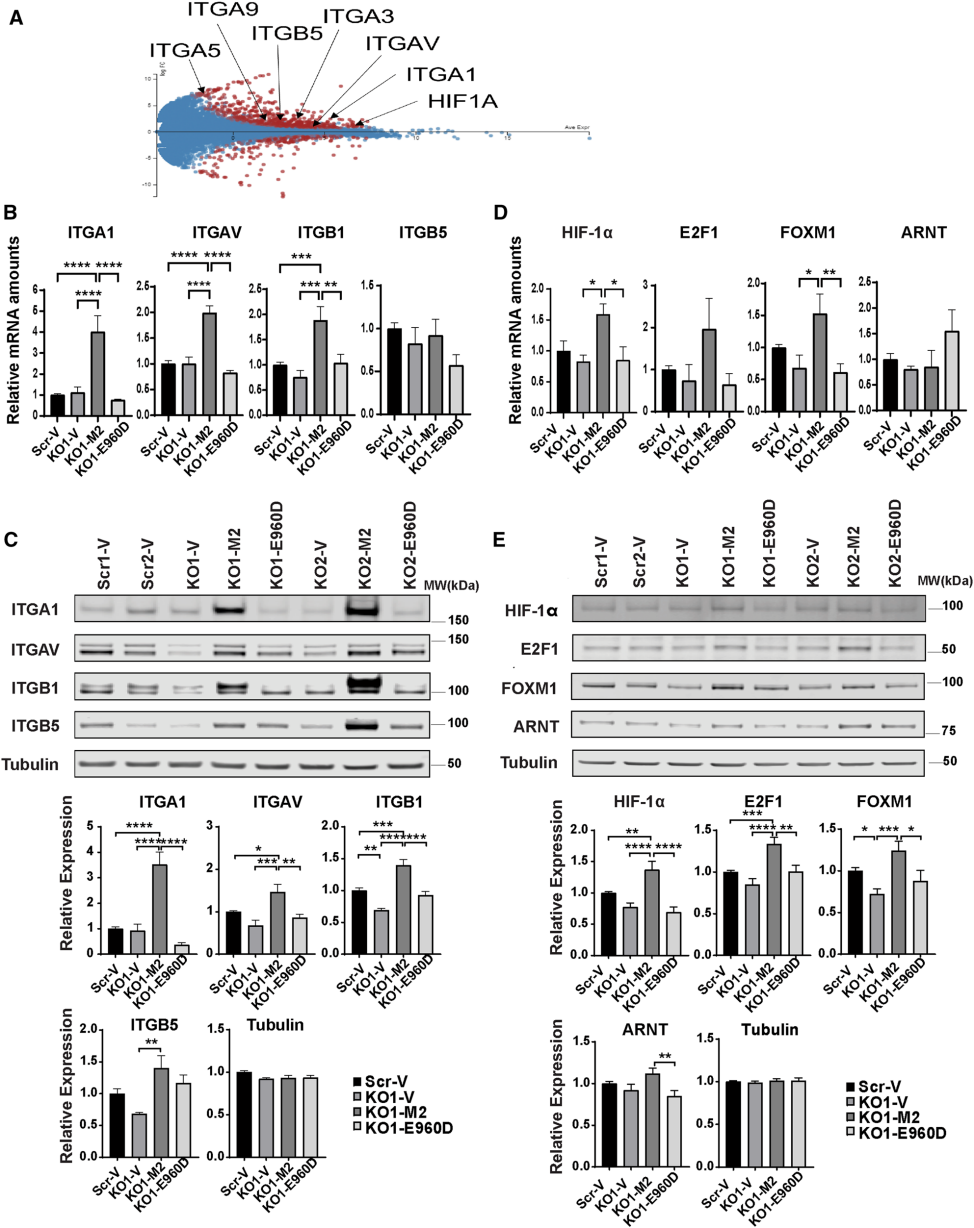
α1, αv, β1, and β5 Integrin expression are increased in TRPM2 reconstituted cells. (**A**) RNA seq analysis of integrin expression in SH-SY5Y cells with TRPM2 deletion (KO-V) compared to the same cells reconstituted with TRPM2 (KO-M2). MA plot (log ratio vs abundance) of RNA seq data is shown. Two biological replicas of each condition were utilized. ITGA1 (α1), ITGA3 (α3), ITGA5 (α5), ITGA9 (α9), ITGAV (αv), and ITGB5 (β5) integrins were significantly increased in KO-M2 cells, as was HIF1A (HIF-1α). These genes with q-value < 0.05 are displayed in red. Positive Log FC indicates genes overexpressed in M2 cells. Degust 4.1.1 software was used for RNA seq analysis and image generation. (**B**), (**D**) RT-qPCR of (**B**) integrins ITGA1, ITGAV, ITGB1, ITGB5, and (**D**) transcription factors HIF-1α, E2F1, FOXM1, and ARNT mRNA from Scr-V, KO1-V, KO1-M2 or KO1-E960D SH-SY5Y cells. RT-qPCR was performed on cells grown without serum for 24 h. Each experimental group was normalized to Scr. Means + S.E.M. of three (ARNT), four (ITGA1, ITGAV, ITGB1, HIF1A), five (E2F1, FOXM1), or seven (ITGB5) experiments performed with KO clone are shown (KO1 in Fig. 3 B, D and a second clone KO2 in Supplementary Fig. S3B,D). Statistics: one-way ANOVA, *p < 0.05, **p < 0.01, ***p < 0.001, ****p < 0.0001. (**C**), (**E**) Western blotting was performed on two clones of TRPM2 KO (KO1-V, KO2-V), KO reconstituted with TRPM2 (KO1-M2, KO2-M2) or E960D (KO1-E960D, KO2-E960D), and scrambled SH-SY5Y control cells (Scr1-V, Scr2-V) grown without serum. Blots were probed with antibodies to (**C**) α1 (ITGA1), αv (ITGAV), β1 (ITGB1), β5 (ITGB5) integrins, or (**E**) transcription factors HIF-1α, E2F1, FOXM1, and ARNT. Tubulin was probed as a control for loading. Densitometry measurements were from five experiments from each clone for each integrin, eight experiments for transcription factors HIF-1α, E2F1, and FOXM1 for KO1 and five experiments for KO2 clone, and eight experiments for ARNT. Results were standardized to each experiment’s scrambled control, and means + S.E.M. for each group are shown. Statistics: one-way ANOVA, *p < 0.05, **p < 0.01, ***p < 0.001, ****p < 0.0001. Results for KO1 are shown in Fig. 3C,E and for KO2 in Supplementary Fig. S3C,E. Full length gels for Western blots are also shown in Supplementary Fig. S3C,E.

To identify mechanisms involved in increased migration and invasion of neuroblastoma cells with high TRPM2 expression, we first examined expression of integrin genes. RNA seq showed significantly increased mRNA for integrins α1, α3, α5, α9, αv, and β5 in neuroblastoma cells highly expressing TRPM2, compared to knockout cells transfected with empty vector (Fig. 3A). Alpha 3, 5, and 9 integrins were not detectable by Western blotting and were not studied further. RT-qPCR and Western blotting were then performed, using cells deprived of serum for 24 h for consistency with migration/invasion conditions. RT-qPCR with primers for α1, αv, or β5 integrins confirmed increased mRNA in cells expressing high levels of TRPM2 (KO1 clone shown in Fig. 3B, KO2 clone in Supplementary Fig. S3B). RT-qPCR also showed an increase in β1 mRNA not detected with RNA seq. These data suggest that increased expression of integrins in cells with TRPM2 high expression is at least in part on a transcriptional basis. Western blotting confirmed significant increases in expression of α1, αv, β1, and β5 integrins in KO cells reconstituted with TRPM2 compared to KO, KO cells reconstituted with E960D, or scrambled control cells (KO1 clone shown in Fig. 3C, KO2 clone in Supplementary Fig. S3C).

### HIF-1α, E2F1, and FOXM1 regulate increased integrin expression in neuroblastoma cells with high TRPM2 expression

The transcriptional basis of increased integrin RNA expression was examined. The ability of transcription factors previously shown to be modulated by TRPM2 inhibition or deletion, including HIF-1α^13^, E2F1^22^, FOXM1^22^, and CREB^23^, to bind to promoter/enhancer regions of these integrins was first studied with GeneCards: The Human Gene Database (https://www.genecards.org). Integrin regulatory domains for HIF-1α/ARNT^48^ were identified in the promoter/enhancer regions of integrin α1 (4 sites), αv (3 sites), β1 (9 sites), and β5 (5 sites). An E2F1 binding site was identified in α1 promoter/enhancer regions, and both E2F1 and FOXM1 binding sites were identified in β1and β5 promoter/enhancer regions. CREB binding sites were also found in the promoter/enhancer regions of all four of these integrins. These data suggest that these transcription factors may play important roles in the increased expression of integrins α1, αv, β1, and β5.

RNA seq was then used to examine differential expression of these transcription factors. Among these, only HIF-1α mRNA was found to be significantly increased by high TRPM2 expression in RNA sequencing analysis (Fig. 3A). Confirming RNA seq results, HIF-1α was determined to be significantly increased in KO cells reconstituted to highly express TRPM2, compared to scrambled control cells or cells in which TRPM2 was deleted, by RT-qPCR or Western blotting (KO1 clone shown in Fig. 3D,E; KO2 in Supplementary Fig. S3D,E). The role of calcium entry was demonstrated because reconstitution of cells in which TRPM2 function was blocked with a calcium impermeant TRPM2 pore mutant did not enhance HIF-1α expression. E2F1 was also shown to be significantly increased in cells with high TRPM2 expression by RT-PCR and Western blotting, compared to cells with TRPM2 deletion or reconstitution with the E960D mutant. In KO1-M2 alone, FOXM1 was statistically increased above KO-V and KO-E960D (Fig. 3D,E; Supplementary Fig. S3D,E). CREB was not increased in KO cells by TRPM2 high expression in these experiments done in serum depleted conditions. Aryl Hydrocarbon Receptor Nuclear Translocator (ARNT) is obligatory for HIF-1α binding to DNA. Although ARNT can be transcriptionally upregulated by HIF-1α^49^, ARNT was not significantly increased in RT-qPCR (Fig. 3D, Supplementary Fig. S3D) or RNA seq analysis. A small increase in ARNT was observed with Western blotting of KO1-M2 (Fig. 3E). These data suggest that increased expression of HIF-1α, E2F1, and FOXM1 together play important roles in increased expression of integrins α1, αv, β1, and β5 in cells with high TRPM2 expression.

### Integrin expression has an important role in TRPM2 modulation of migration and invasion

**Figure 4:**
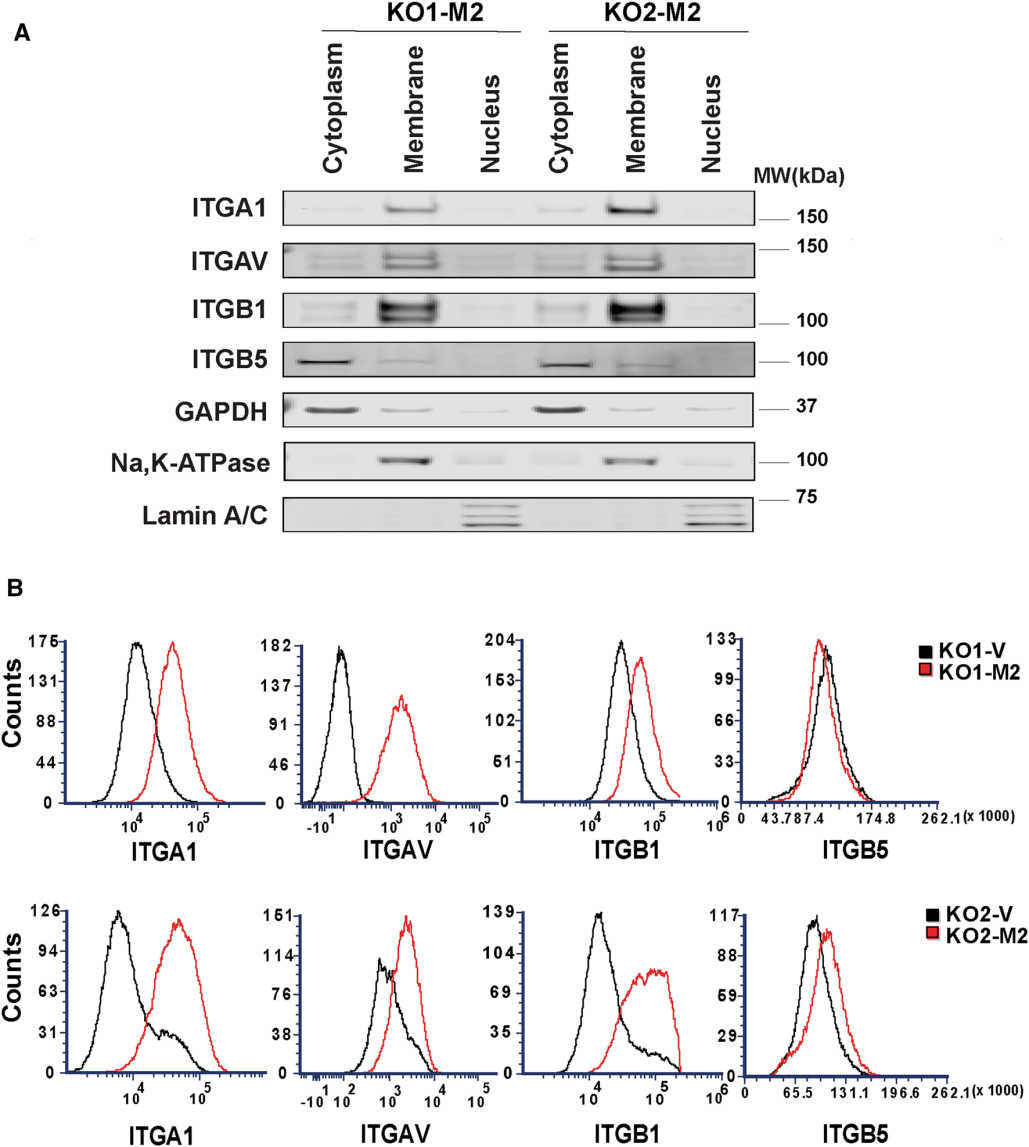
Subcellular Fractionation of α1, αv, β1, and β5 integrins in neuroblastoma cells highly expressing TRPM2. (**A**) Subcellular separation of two clones of SH-SY5Y KO cells reconstituted with TRPM2 (KO1-M2, KO2-M2) into cytoplasmic, membrane, and nuclear fractions was performed as described in "Materials and Methods". Western blotting was performed with fractionated samples loading equivalent amounts per lane (10 ug/lane). Blots were probed with antibodies to α1 (ITGA1), αv (ITGAV), β1 (ITGB1), β5 (ITGB5) integrins. Blots were also probed with antibodies to GAPDH (cytoplasmic marker), Na,K-ATPase α (plasma membrane marker which regulates Na and K ions and cell volume) and Lamin A/C (nuclear marker) as controls. Four experiments were performed with similar results and the results of one are shown. α1, αv, and β1 were predominantly found in the membrane fraction, and β5 in the cytoplasmic. Full length gels for these Western blots are shown in Supplementary Fig. S4. Membrane fractionations of Scr1-V, Scr2-V, KO1-V, KO2-V, KO1-M2, KO2-M2, KO1-E960D, KO2-E960D cells are also shown on Supplementary Fig. S4. (**B**) Flow Cytometry of α1, αv, β1, and β5 integrin expression on the surface of non-permabilized SH-SY5Y KO cells (KO1-V, KO2-V) and KO cells expressing TRPM2 (KO1-M2, KO2-M2). Fluorescent intensities (X-axis) of integrins α1, αv, and β1 were increased on the surface of KO-M2 compared to KO-V cells. This experiment was performed twice with similar results and results of one experiment are shown.

**Figure 5:**
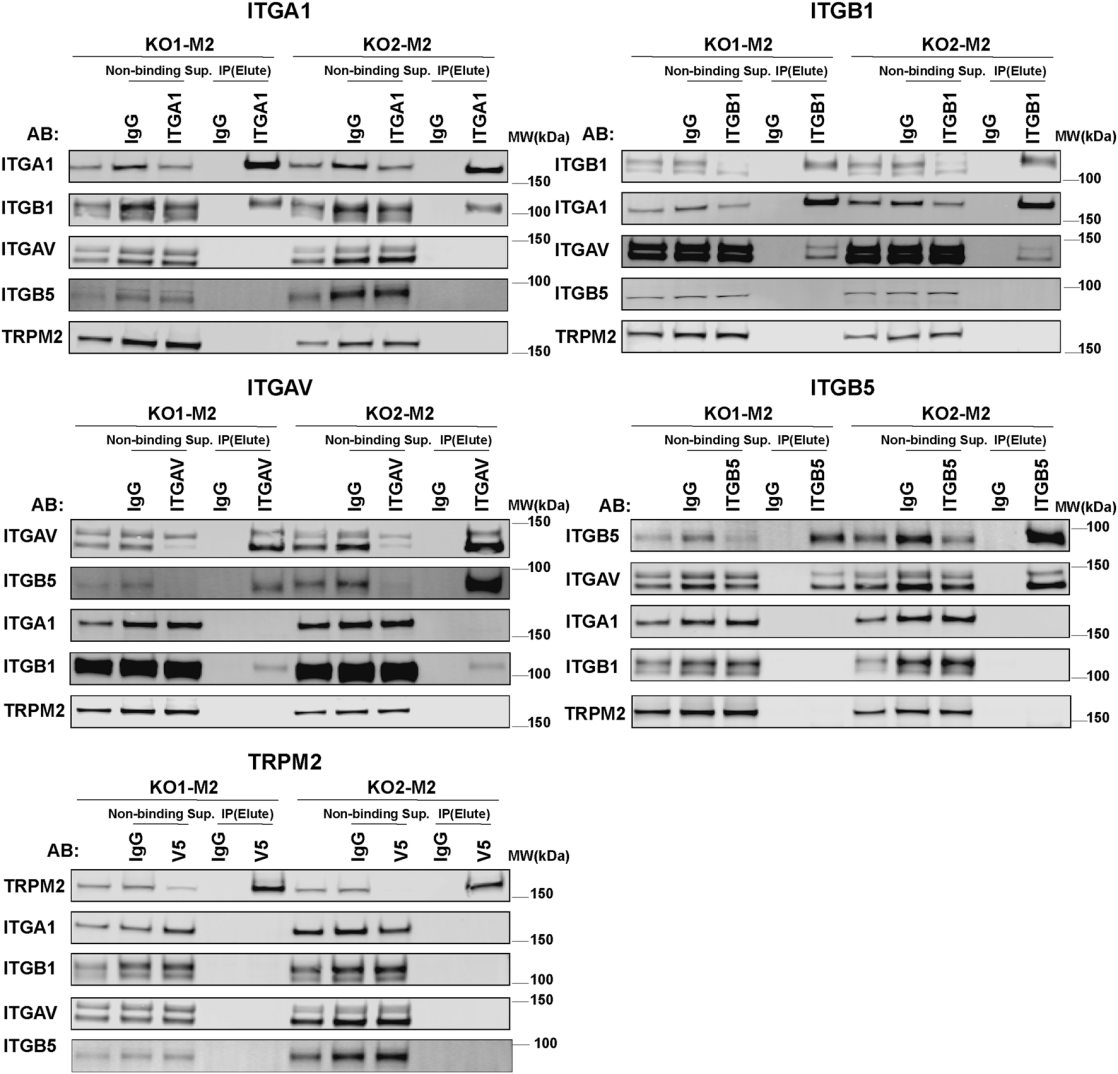
Immunoprecipitation of integrin complexes in TRPM2 expressing cells. Integrins α1 (ITGA1), αv (ITGAV), β1 (ITGB1), β5 (ITGB5) were immunoprecipitated (IP) from SH-SY5Y cells with TRPM2 deletion reconstituted with TRPM2, grown without serum for 24 h, with each integrin antibody, or nonspecific IgG. Western blotting of immunoprecipitates was performed on nonbinding supernatants and eluates of bound proteins with the same antibodies. Anti-V5 antibody was used to immunoprecipitate V5-labeled TRPM2, and anti-TRPM2 C-terminal antibody was used for Western blotting. A sample of cell lysate (20 µg/15 µl) was loaded in the first lane to demonstrate starting proteins, and 30 µg/15 µl was loaded in non-binding supernatant lanes. Two experiments were performed with each antibody. Integrin α1 co-precipitated reciprocally with β1, and αv coprecipitated reciprocally with β5 and β1. Representative results are shown. None of the integrins co-precipitated with TRPM2. Full length gels for Western blots are shown in Supplementary Fig. S5.

To determine the intracellular localization of integrins α1, αv, β1, and β5, which are highly expressed in cells with increased TRPM2 expression, subcellular fractionation of SH-SY5Y KO cells reconstituted with TRPM2 was performed. The predominant localization of α1, αv, and β1 integrins was in the membrane fraction (Fig. 4A), where they are functionally most active. Integrin β5 was predominantly found in the cytoplasm, and was present in the membrane in small quantities compared to the other integrins. Subcellular fractionation of Scr, KO, and KO-E960D cells was also performed, but in these cells with lower integrin expression compared to KO-M2, much less membrane expression of integrins was observed except for β1 integrin (Supplementary Fig. S4). Next, we performed flow-cytometry analysis of non-permeabilized cells to examine cell surface integrin subunits (Fig. 4B). Consistent with the subcellular fractionation results, KO1-M2 and KO2-M2 cells had greater cell surface expression of α1, αv, and β1 integrins compared to KO cells, whereas β5 was minimally increased in the plasma membrane of KO-M2 cells, probably because its location is primarily cytoplasmic (Fig. 4A). Integrins form functional complexes which are heterodimers consisting of an alpha and beta subunit associated by non-covalent interactions. To determine the integrin complexes that are present in these neuroblastoma cells, coimmunoprecipitation with integrin antibodies followed by Western blotting was performed (Fig. 5). Integrin α1 immunoprecipitated strongly only with β1. Integrin β1 immunoprecipitated strongly with α1 and weakly with αv. Integrin αv precipitated with β5 strongly and weakly with β1. Integrin β5 immunoprecipitated strongly with αv. None of the integrins co-precipitated with TRPM2. These data demonstrated that the predominant integrin complexes in KO-M2 cells are α1β1 and αvβ5, and with weaker presence of αvβ1.

**Figure 6:**
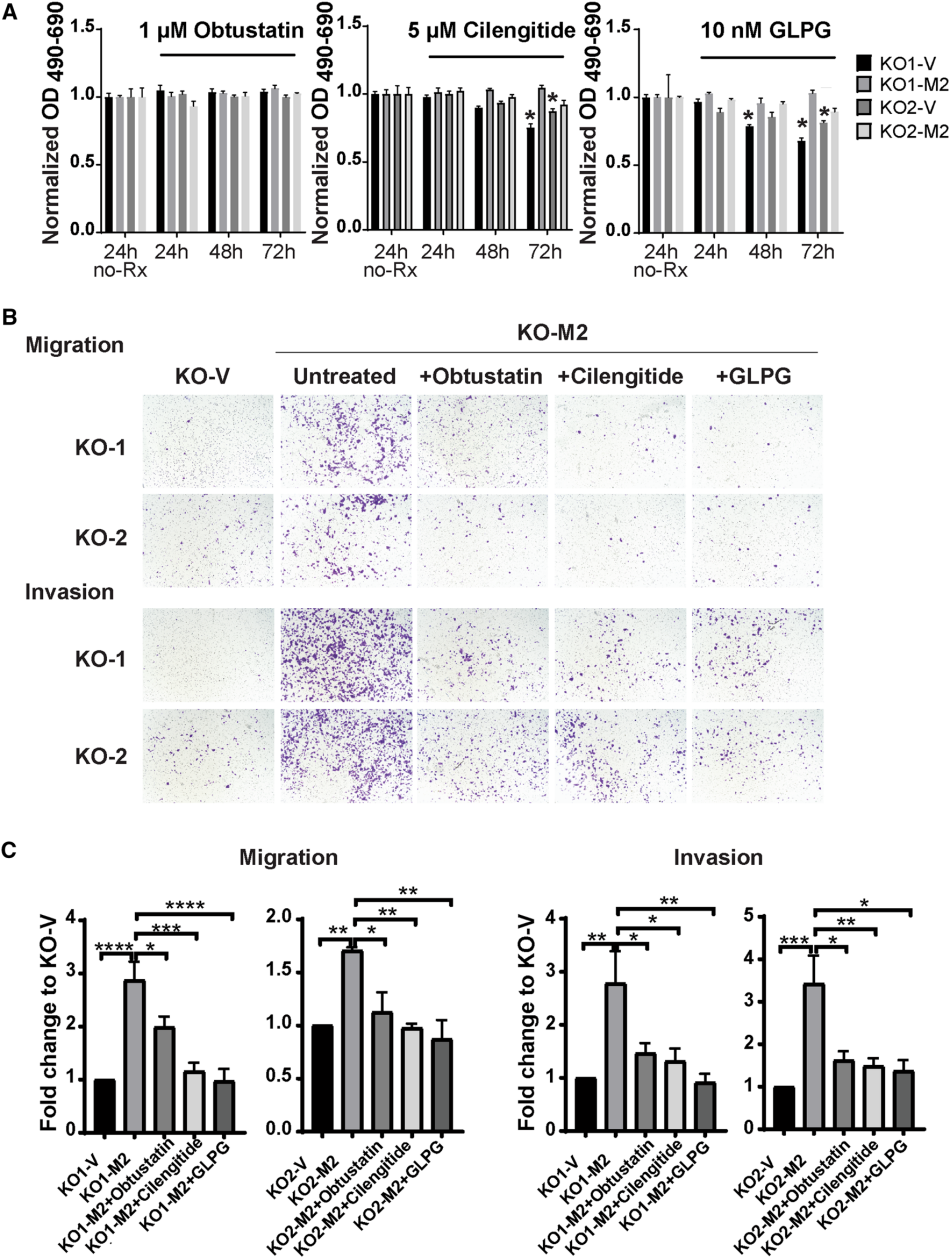
Integrin antagonists block increased migration and invasion found in neuroblastoma cells with high TRPM2 expression. Two clones of SH-SY5Y knockout cells (KO1-V, KO2-V) and TRPM2 KO cells reconstituted to express TRPM2 (KO1-M2, KO2-M2) were untreated or treated with integrin complex antagonists obtustatin (α1β1 inhibitor), cilengitide (αvβ5 inhibitor), or GLPG-0187 (αvβ1 and αvβ5 inhibitor). (**A**) Cell viability was examined with XTT analysis following treatment of cells with obtustatin (1 µM), cilengitide (5 µM) or GLPG-0187 (10 nM) for 24, 48, or 72 h, the concentrations used in invasion and migration studies. Results were normalized to untreated cells at each time point for each clone (n = 4 replicates). Only the 24 h untreated (no-Rx) normalization is shown here. Three similar experiments were performed and means + S.E.M. of one are shown. Results were analyzed with two-way ANOVA. Viability of treated KO-M2 cells was not significantly reduced compared to untreated cells. *p < 0.05 indicates a reduction seen in KO-V cells, consistent with their increased sensitivity to reduced viability. In Supplementary Fig. S6A, viability after treatment of KO1,2-V and KO1,2-M2 cells with additional doses of obtustatin (0.5, 1 µM), cilengitide (1 µM, 5 µM) or GLPG-0187 (5, 10 nM) at 24, 48, or 72 h are shown. In Supplementary Fig. S6B, viability after treatment of Scr1,2-V, KO1,2-V, KO1.2-M2 and KO1,2-E960D cells with obtustatin (1 µM), cilengitide (1 µM, 5 µM) or GLPG-0187 (10, 20 nM) at 24 or 48 h are shown. (**B**), (**C**) Migration and invasion assays were performed as described in "Materials and Methods" and representative pictures from Boyden chambers are shown (**B**). Analysis of migration of two clones of KO and KO-M2 cells in three experiments and invasion density in five experiments was performed and results shown in (**C**). Two clones each of untreated KO-V (KO1-V or KO2-V), untreated KO-M2 (KO1-M2 or KO2-M2), or KO-M2 cells treated with obtustatin (1 µM), cilengitide (5 µM), or GLPG-0187 (10 nM) were studied for 48 (migration) or 72 h (invasion). Mean + S.E.M. density is shown for three migration and five invasion experiments. Results for each well in each experiment were standardized to each clone’s KO-V, and the mean number for each clone in each experiment (3 to 4 replicates) was used to calculate the mean + S.E.M. from all experiments. Statistical differences of each group compared to its KO-V were analyzed by one-way ANOVA, *p < 0.05, **p < 0.01, ***p < 0.001, ****p < 0.0001.

To determine the importance of these integrins complexes in the increased migration and invasion found in cells with high TRPM2 expression, migration and invasion assays were performed after incubation of cells with the inhibitors obtustatin (α1β1 antagonist), cilengitide (αvβ5 antagonist), and GLPG-0187 (αvβ1 and αvβ5 antagonist). These three inhibitors had minimal effect on cell viability in the doses used here (Fig. 6A, Supplementary Fig. S6A,B). When effects on viability were seen, they were usually in KO clones, which previously have been shown to be more sensitive to other agents including doxorubicin^14,19,23^. Each inhibitor significantly reduced migration and invasion in TRPM2 reconstituted cells, suggesting that all of these integrin complexes contribute to increased metastatic potential (Fig. 6B,C).

### Akt and ERK activation also promote migration in neuroblastoma cells highly expressing TRPM2

**Figure 7:**
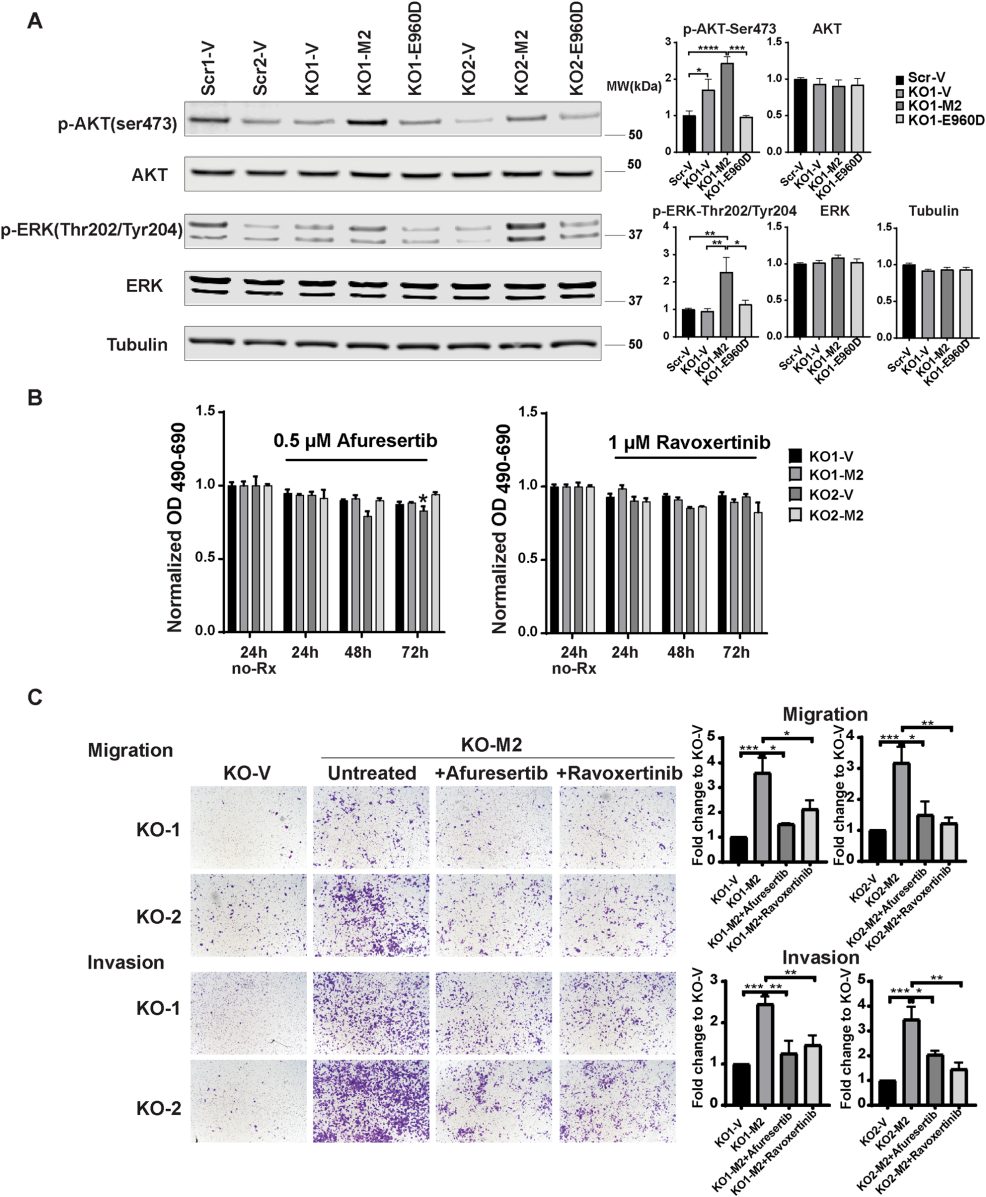
Akt and ERK activation promote migration and invasion in cells highly expressing TRPM2. (**A**) Western blots of lysates from SH-SY5Y KO cells (KO1-V, KO2-V), KO cells stably expressing TRPM2 (KO1-M2, KO2-M2), E960D (KO1-E960D, KO2-E960D), or scrambled control cells (Scr1-V, Scr2-V) were probed with antibodies to pAkt^Ser473^, Akt, pERK^Thr202/Tyr204^, ERK, and tubulin in four experiments. One blot is shown for each antibody. Densitometry measurements were standardized to scrambled control for each blot. Means + S.E.M. for each group are shown for four experiments (KO1 clone shown in Fig. 7A, KO2 clone shown in Supplementary Fig. S7A). Statistics: one-way ANOVA, *p < 0.05, **p < 0.01, ***p < 0.001. Full length gels for Western blots shown in Supplementary Fig. 7A. (**B**) Cell viability was examined with XTT analysis following treatment of cells with the Akt inhibitor afuresertib (0.5 uM) or the ERK inhibitor ravoxertinib (1 uM) for 24, 48, or 72 h. Three experiments were performed with each inhibitor and results of one are shown in Fig. 7B and another in Supplementary Fig. S6C. Results were normalized to untreated cells (no-Rx) at each time point for each clone (n = 4 replicates). Only the 24 h untreated (no-Rx) normalization is presented in Fig. 7B. Means + S.E.M. are shown and results analyzed with two-way ANOVA. KO-M2 cell viability was not significantly reduced compared to untreated cells. *p < 0.05 indicates a reduction in KO-V cells. Treatment with additional doses of Akt inhibitor afuresertib (0.1, 0.5 uM) or the ERK inhibitor ravoxertinib (0.5, 1 uM) for 24, 48, or 72 h are shown in Supplementary Fig. S6C. (**C**) Migration and Invasion assays of two clones each of SH-SY5Y KO cells or KO cells stably expressing TRPM2, untreated or treated with the Akt inhibitor afuresertib (0.5 uM) or the ERK inhibitor ravoxertinib (1 uM). Migration and invasion assays were performed as described in "Materials and Methods" and representative pictures from Boyden chambers are shown. Analysis of migration and invasion density was performed. Three experiments were performed with 0.5 µM afuresertib for inhibition of migration and invasion. For I µM ravoxertomib, five experiments were performed for migration, and four experiments were performed for invasion. Results of individual wells were standardized to average KO-V in each group in each experiment. Mean + S.E.M. for each experiment was determined for untreated KO1,2-V, untreated KO1,2-M2, and KO1,2-M2 cells treated with afuresertib or ravoxertinib. The mean from each experiment was used to calculate the mean + S.E.M. shown. Statistical differences among groups were analyzed by one-way ANOVA. *p < 0.05, **p < 0.01, ***p < 0.001, ****p < 0.0001.

Akt has a role in promotion of migration and invasion in cancer through mechanisms including upregulation of β1 expression^50–53^ and activation of integrins including β1 and αvβ5^54^. Akt is activated by TRPM2^34^. ERK, taken here to indicate ERK1 and ERK2, has roles in regulation of migration involving integrins^55–57^. ERK also activates β1 integrin by enhancing its sialylation^58^. The roles of Akt and ERK phosphorylation in enhanced migration and invasion mediated by TRPM2 in neuroblastoma were examined here. Akt and ERK expression were not significantly different between TRPM2 KO cells, KO cells reconstituted with TRPM2, or scrambled controls. However, phosphorylation of Akt at serine 473 and ERK at threonine202/tyrosine 204 were greater in KO cells reconstituted with TRPM2 compared to KO-V, KO-E960D, or scrambled control cells (KO1 clone shown in Fig. 7A; KO2 in Supplementary Fig. S7A). The role of Akt and ERK in increased migration or invasion of neuroblastoma cells with high TRPM2 levels was examined following treatment with the Akt inhibitor afuresertib or the ERK inhibitor ravoxertinib. Neither of these inhibitors significantly affected viability of cells highly expressing TRPM2 at the doses used (Fig. 7B, Supplementary Fig. S6C). Inhibitors of Akt and ERK blocked the increased migration and invasion observed in cells overexpressing TRPM2 (Fig. 7C), confirming their role in this pathway.

### Depletion of Endogenous TRPM2 in SK-N-AS neuroblastoma cells significantly reduces migration and invasion

**Figure 8:**
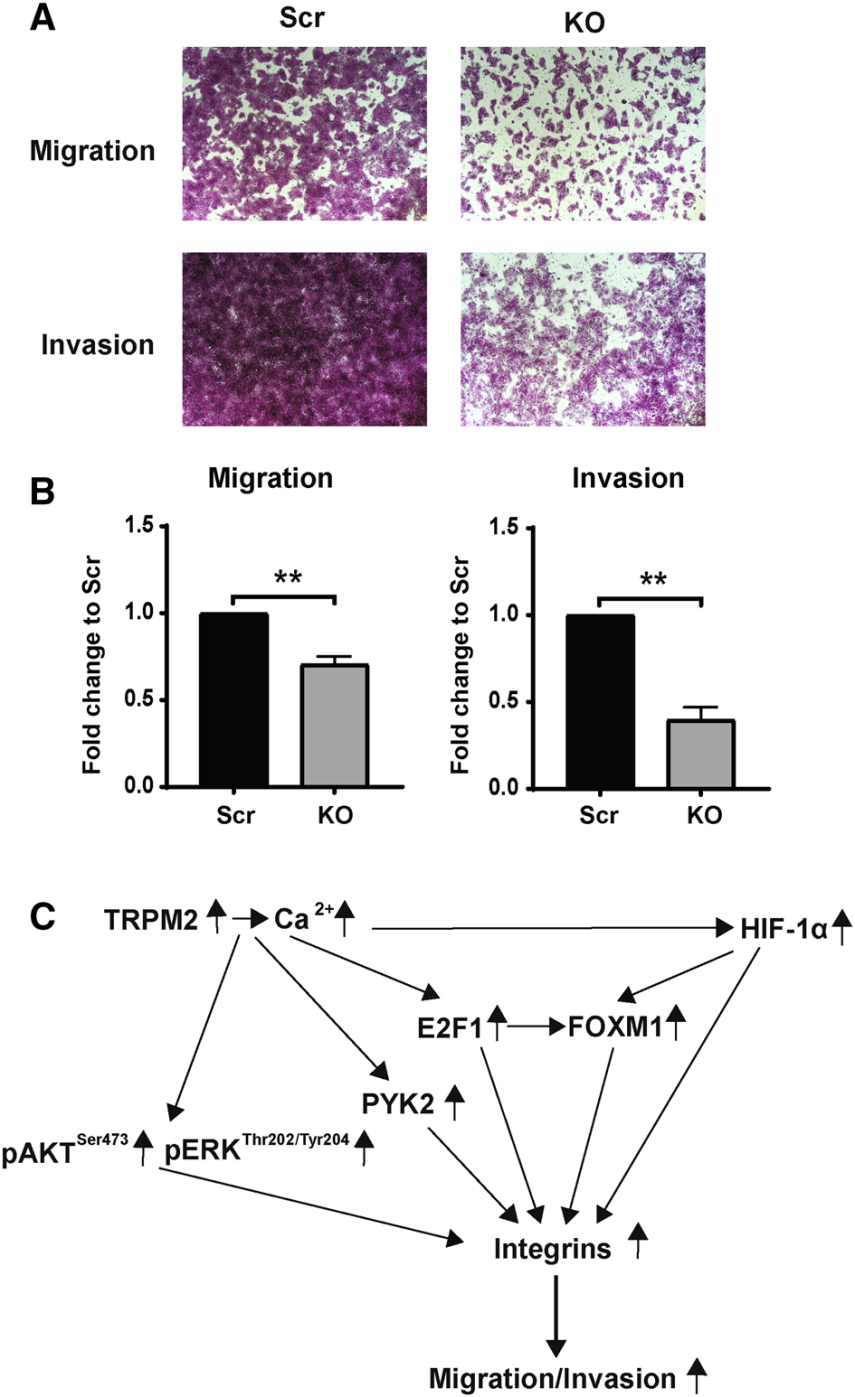
Migration and Invasion in neuroblastoma cells. (**A**), (**B**) Migration and invasion assays were performed with SK-N-AS KO cells (KO) and scrambled control cells (Scr-V) as described in Methods. Representative pictures from Boyden chambers of one migration and invasion experiment are shown in (**A**). Pictures from two additional migration and invasion experiments with SK-N-AS cells are shown in Supplementary Fig. 8C. (**B**) Densitometry analysis of migration and invasion density of scrambled and TRPM2 KO SK-N-AS cells. Three migration experiments and three invasion experiments were performed. The mean from each experiment was quantitated comparing KO to scrambled cells, and used to calculate mean + S.E.M. of all three. Statistical difference of SK-N-AS KO compared to scrambled control was analyzed by two-tailed T-test, **p < 0.003. (**C**) Schema of TRPM2 modulation of migration/invasion in neuroblastoma. Higher levels of TRPM2 enhance migration and invasion. In SH-SY5Y cells, increased migration and invasion were associated with increased expression of HIF-1α, E2F1, and FOXM1 transcription factors and downstream integrin targets, and activation of Akt and ERK.

SH-SY5Y cells have low levels of migration and invasion, which are significantly greater when the level of endogenous TRPM2 is increased. To examine the effect of endogenous TRPM2 depletion on migration and invasion, SK-N-AS cells, which are an aggressive neuroblastoma cell line with high levels of migration and invasion^59^, were studied. TRPM2 depletion utilized CRISPR-Cas9 technology as described in "Materials and Methods". Depletion of TRPM2 in SK-N-AS KO cells was shown with RT-qPCR (Supplemental Fig. S8A). Calcium entry was stimulated by hydrogen peroxide in wild type and scrambled control SK-N-AS cells, but was significantly reduced in TRPM2 KO cells (Supplementary Fig. S8B). In SK-N-AS KO cells, migration and invasion were significantly reduced compared to scrambled controls (Fig. 8A,B; Supplementary Fig. S8C). These studies confirm previously published results in pancreatic ductal adenocarcinoma cells^35^ and in gastric cancer cells^34^, that reduced levels of endogenous TRPM2 can down modulate migration and invasion. In gastric cancer, integrins were also decreased after reduction in endogenous TRPM2.

## Discussion

TRPM2 is a cation channel which is highly expressed in many cancers^2,12,15,18,60^. It plays key roles in modulating cell proliferation and survival through maintenance of mitochondrial function^13,14^, bioenergetics^19^, and antioxidant response^24^. TRPM2 is also involved in cell cycle and DNA damage repair^22^. Many of these functions are regulated through modulation of transcription factors including HIF-1α^13,19^, E2F1^22^, FOXM1^22^, CREB^23^, and Nrf2^19,24^. TRPM2 has recently been reported to regulate migration and invasion^34,35^, essential components in development of metastasis. Here, the role of TRPM2 in migration and invasion of neuroblastoma and the mechanisms were investigated. High TRPM2 expression significantly enhanced expression of α1, αv, β1 and β5 integrins in the membrane of neuroblastoma cells and integrin complex formation, through transcriptional mechanisms including increased HIF-1α, E2F1, and FOXM1, resulting in promotion of migration and invasion.

High TRPM2 levels correlate with worse patient outcome in pancreatic cancer^35^, and gastric cancer, particularly at advanced stage^17^. Here, high TRPM2 expression was associated with significantly poorer outcome in Stage 4 non-MYCN amplified neuroblastoma patients using three publicly available databases^40–42^. This was not observed in Stage 4 MYCN amplified patients, possibly because of activation of other oncogenic targets by MYCN including FOXM1^43^. In our studies, high TRPM2 expression in neuroblastoma cells resulted in increased migration and invasion, essential features of metastatic disease. Migration and invasion were reduced in cells with lower TRPM2 expression and in TRPM2 KO cells. The amount of migration between cell types, for example SH-SY5Y vs SK-N-AS, also varied, indicating that TRPM2 is a modifier of migration and invasion among other determinants. RNA sequencing, RT-qPCR, and Western blotting of samples from cells with high TRPM2 all demonstrated significantly elevated α1, αv, β1, and β5 integrin expression and high membrane expression of α1, αv, and β1.

The involvement of integrins in many stages of tumor progression, from primary tumor development involving oncogenic growth factor receptor signaling to cancer migration/invasion and colonization of metastatic sites, has recently been reviewed^61^. Integrin activation and binding to the extracellular matrix triggers recruitment of a complex array of scaffold and cytoskeletal proteins, which regulate normal and malignant processes. In cancer, different integrins are involved in the multiple steps of cancer cell migration, invasion into vasculature, survival of circulating tumor cells, and colonization of metastatic sites. In SH-SY5Y neuroblastoma cells with increased expression of TRPM2, integrin complexes of α1β1, αvβ1 and αvβ5 were identified. Each integrin has distinct ligand-binding specificity and distribution in tissues^62,63^. Integrin α1β1 is highly expressed in many cancers including lung, breast, and colorectal^64^, and the α1β1 complex is key to the ability of cells to contact and migrate in collagen. In addition, αv increases adhesion and migration and increases brain metastasis in a number of solid tumors^65^, and complexes αvβ1 and αvβ5 bind with ligands vitronectin and fibrinogen^66^. β1 integrin expression and activation have a key role in metastasis formation^36,37,67^. In pancreatic cancer, knockdown of β1 integrin inhibited adhesion, migration, primary tumor growth, and metastasis^37^. In breast cancer, β1 integrin critically modulated vascular adherence and transendothelial migration^61,68^. Here, inhibitors of each of the three complexes α1β1, αvβ1, and αvβ5 significantly inhibited the increased migration and invasion in neuroblastoma cells highly expressing TRPM2. This observation suggests that the mechanisms by which these integrin complexes mediate migration and invasion are not redundant, i.e. one cannot compensate for the loss of another.

RNA sequencing analysis and RT-qPCR demonstrated that integrins α1, αv, β1, and β5 are significantly increased in cells expressing high levels of TRPM2 on a transcriptional basis. A role for HIF-1α and ARNT in high integrin expression associated with increased TRPM2 levels was predicted based on the number of HIF-1α/ARNT binding sites in the promoter and enhancer regions of all four of these integrins (GeneCards The Human Gene Database; four for integrin α1, three for integrin αv, nine for integrin β1, and five for integrin β5). HIF-1α and its downstream targets are increased by TRPM2 expression^13^. The mechanism of modulation of HIF-1α expression in SH-SY5Y cells with higher TRPM2 involved increased HIF-1α transcription and reduced von Hippel Landau expression, consistent with reports that HIF-1α transcription can be induced by calcium ionophores. HIF-1α in turn also regulates genes involved in glycolysis, oxidative stress, and angiogenesis^13^. Here, elevation of HIF-1α mRNA and protein in cells highly expressing TRPM2 supports the hypothesis that it transcriptionally contributes to the increased expression of α1, αv, β1 and β5 integrins, and the increase in migration and invasion (Fig. 3). HIF-1α was previously shown to be required for β1 integrin expression^39^, and α1 and β1 expression correlated with a HIF-1 expression signature^69^. Hypoxia also regulates av integrin and its transcription in endothelial cells^70^ and cell surface expression in melanoma^71^.

TRPM2 deletion reduces expression of the transcription factors HIF-1α^13^, E2F1^22^, FOXM1^22^, and CREB^23^, raising the possibility that a significant increase in TRPM2 may modulate integrins through an increase in their expression. FOXM1 is a master regulator in cancer^25^ and promotes cancer progression through many pathways including key roles in tumor proliferation, cell cycle progression, DNA damage repair, angiogenesis, and drug resistance^25,26^. Inhibition of FOXM1 results in decreased proliferation, DNA repair, migration, and metastasis^29^, whereas elevated FOXM1 is associated with a worse prognosis in many cancers^30^. E2F1 is also a master regulator in cancer^72,73^. For integrin α1, E2F1 binds to one promoter/enhancer site; for β1, E2F1 binds to two sites and FOXM1 to eight sites; for β5, E2F1 and FOXM1 each bind to one site. Increased E2F1 and FOXM1 in cancers with high levels of TRPM2 may contribute to transcriptionally increased expression of α1, β1 and β5 integrins, and greater migration and invasion. Increased TRPM2 in tumors may be both a driver of high FOXM1 and the embryonic stem cell program seen in non-MYCN amplified Stage 4 neuroblastoma patients, contributing to increased metastasis and worse outcome^43^. Although binding sites for CREB were identified in all four integrin promoter/enhancer regions, significantly increased CREB expression was not found here in cells with high TRPM2 expression. Increases in CREB5 and CREBRF were observed with RNA seq, raising the possibility that other proteins in the CREB pathway could promote metastasis^74,75^. These data suggest that TRPM2 mediates integrin expression and migration/invasion of neuroblastoma through modulation of HIF-1α, E2F1, and FOXM1.

High TRPM2 levels may contribute to increased migration and invasion in neuroblastoma cells through a number of mechanisms involving increased calcium entry and/or mitochondrial dynamics^32^, in addition to enhanced integrin expression (Fig. 8C). One pathway involves Akt and ERK signaling^50–52,76^. The PTEN/Akt signaling pathway is also required in TRPM2-modulated migration and invasion of gastric cancer cells^34^. In these cells, after knockdown of TRPM2, treatment of cells with the Akt inducer Sc79 increased Akt phosphorylation, rescued cell motility, and restored migration and invasion, through mechanisms including epithelial-mesenchymal transition (EMT)^34^. ERK is a master regulator of many fundamental cellular processes including cell proliferation, survival, metabolism, and migration^56,57^ through mechanisms including Src and EGF/EGFR activation^55,77^ and maintenance of slug, a cell mobility inducer. The pathways involving Akt and ERK in integrin modulation are complex because integrins themselves modulate Akt and ERK activation; β1 integrin can induce phosphorylation and activation of Akt^78^, and αv and other integrins induce activation of ERK^79,80^. Here, we demonstrate that in neuroblastoma cells highly expressing TRPM2, phosphorylation of both Akt and ERK are increased and inhibitors of Akt and ERK decreased migration and invasion. These pathways may play a role in the increased migration and invasion of these cells. Expression and phosphorylation of the kinase Pyk2 were previously shown by our laboratory to be increased in cells highly expressing TRPM2^23^. Pyk2 has roles in metastasis including integrin β1 stabilization, which could contribute to increased β1 protein reported here^81^. When malignant cells circulate, as shown in melanoma, they experience high levels of oxidative stress, and successfully metastasizing cells are predicted to have higher antioxidant capacities which allow them to survive^82,83^. High TRPM2 levels may also promote metastasis through the demonstrated ability of TRPM2 to increase anti-oxidant capacity through greater expression of Nrf2, antioxidant cofactors, and superior bioenergetics^24^. Integrins and ion channels have complex functional and physical interactions^84,85^. The possibility that an interaction between TRPM2 and integrins has a role in increased migration or invasion in neuroblastoma was considered, but in our model, integrins with elevated expression did not co-immunoprecipitate with TRPM2.

Oxidative stress, which is present in many tumors, has been shown to cause an increase in migration, invasion, and tumor extravasation. Among pathways activated by oxidative stress, ADPR is increased through mechanisms including DNA damage by ROS and NADase mediated ADPR production. Increased ADPR in turn activates TRPM2 by binding to its C-terminus, promoting calcium entry, which results in expression of genes involved in migration and invasion. This work demonstrates that high TRPM2 expression in neuroblastoma has a functional role in increasing migration and invasion through enhanced expression of α1, αv, β1, and β5 integrins and integrin complexes (Figs. 4, 5, 8C). The increase in integrin expression is calcium dependent. For α1, αv, β1, and β5, the increase is at least in part on a transcriptional basis and associated with greater levels of transcription factors HIF-1α, E2F1, and FOXM1 in cells expressing high TRPM2, which bind to sites in integrin promoter/enhancer regions. Activation of Akt and ERK in cells with high TRPM2 expression also modulates increased neuroblastoma migration and invasion. The combined effects of high TRPM2 expression on increasing migration and invasion, shown here, as well as enhancing cell survival through maintenance of mitochondrial function, cellular bioenergetics, antioxidant response, DNA repair and promotion of cell cycle progression, shown previously, strongly support the targeting of TRPM2 in neuroblastoma and in a number of cancers, which would impact proliferation, survival, and metastasis.

## Materials and methods

### Deletion of TRPM2 with CRISPR and Generation of stably transfected neuroblastoma cell lines

The neuroblastoma cell line SH-SY5Y was obtained from the American Type Culture Collection (ATCC, Manassas, Va, USA). SH-SY5Y cells were cultured in 50% DMEM and 50% Ham’s F-12 (DMEM/F-12 50/50) media supplemented with 10% heat-inactivated FBS^13,19^; cells used in RT-qPCR and Western blotting were serum deprived for 24 h prior to collection. TRPM2 knockout (KO) and scrambled control SH-SY5Y neuroblastoma cells were generated in the Miller laboratory with CRISPR technology and cultured as described previously^19^. TRPM2 genomic DNA encoding the first 40 amino acids were deleted and the remaining TRPM2 sequence was frameshifted. RT-PCR and Western blotting of TRPM2 confirming TRPM2 depletion was published previously^19^. Scrambled (Scr) control cells used in experiments were generated following the CRISPR protocol except that they were exposed to scrambled gRNA instead of TRPM2 targeted. The E960D construct was created using wild type TRPM2 in pcDNA3.1V5/His vector as a template, Quick Change kit (Stratagene) and the following primers: forward 5’-CTCATCCACAACGACCGCCGGGTGGAC-3’, reverse 5’-TCCACCCGGCGGTCGTTGTGGA TGAG-3’ as described^19^. In TRPM2 reconstitution experiments, SH-SY5Y KO cells were transfected with wild type TRPM2, TRPM2 E960D mutant^44,45^, or empty vector using the Neon Transfection System as described^19^. Inability of the TRPM2 calcium impermeant mutant E960D to gate calcium entry has been established in HEK cells and in global TRPM2 KO cardiac cells transfected with wild type TRPM2 compared to the E960D mutant^44,45^. SK-N-AS cells are an aggressive neuroblastoma cell line derived from bone marrow metastasis and obtained from the ATCC. SK-N-AS cells with knockout of TRPM2 and scrambled control cells were generated by Biocytogen (Beijing, China) using EGE(CRISPR-Cas9) gene editing to remove part of TRPM2 exon 3 and exon 4. After deletion of exon 4, the remaining exons were out of frame. TRPM2 depletion in KO cells was confirmed by RT-qPCR (Supplementary Fig. S8A).

### Bioinformatics analysis of TRPM2 expression and neuroblastoma survival

Gene Expression Profiling Interactive Analysis 2 (GEPIA2)^86^ was used to assess the levels of TRPM2 in public databases comparing normal and tumor tissues. Data from normal tissues were obtained from The Cancer Genome Atlas (TCGA) Research Network (https://www.cancer.gov/tcga) and Genotype-Tissue Expression (GTEx) datasets, while tumor tissues were obtained from the TCGA dataset. These datasets are publicly available. All the procedures were performed in accordance with the relevant guidelines and regulations. The expression of TRPM2 in all 32 cancer types available at GEPIA2 was compared to normal samples. One-way ANOVA was used for the statistical analysis and pairs with a |Log2FC|> 1 and *p* value < 0.01 were reported. For the analysis of neuroblastoma samples, the R2 platform^87^ was used. The expression of TRPM2 was analyzed in the three datasets available with the highest number of neuroblastoma samples: Cangelosi^40^ (786 samples), Kocak^41^ (649 samples) and Westermann (579 samples). In each dataset, the expression levels of TRPM2 were compared between MYCN-amplified and non-MYCN amplified samples across neuroblastomas at all stages or only Stage 4. Statistical analysis was performed with unpaired t-test (**p < 0.01, ***p < 0.001, ****p < 0.0001). Survival plots of Stage 4 neuroblastoma without MYCN amplification were performed using TRPM2 expression levels as grouping criteria. Samples with TRPM2 expression at the last quartile were classified as High TRPM2 and the remaining samples were classified as Low TRPM2. Two datasets and analysis software from the R2 platform were used for survival analysis: Cangelosi^40^ with 198 Stage 4 non-MYCN amplified samples and Seeger^42^ with 102 samples. Statistics were performed with one-way ANOVA.

### RNA Seq

RNA from SH-SY5Y cells with TRPM2 deletion (KO) or KO reconstituted with TRPM2 was prepared using RNeasy kit (Qiagen, Hilden, Germany) and analyzed by the PSCOM Genomic Science Core Facility. Differential expression analysis between these two conditions (two biological replicates per clone) was performed using the EdgeR package. The resulting P values were adjusted using Benjamini–Hochberg to control the false discovery rate (FDR or q-value). Genes with an adjusted P-value (FDR or q-value) < 0.05 found by EdgeR were assigned as differentially expressed. The RNA seq data for SH-SY5Y cells discussed in this publication are deposited in NCBI’s Gene Expression Omnibus^46,47^ and are accessible through GEO Series accession number GSE203660. Degust 4.1.1 (http://degust.erc.monash.edu/) software, a web-tool for RNA seq analysis, was used to generate Fig. 3A.

### RT-qPCR

RNA was prepared from TRPM2 depleted (KO) SH-SY5Y cells, TRPM2 KO cells reconstituted with TRPM2 or the calcium impermeant TRPM2 mutant E960D, or scrambled (Scr) control cells using the RNeasy kit (Qiagen, Hilden, Germany). Cells were cultured without serum for 24 h prior to harvest to simulate migration/invasion conditions. First-strand cDNA synthesis was performed from 2000 ng of RNA using Super Script kit (Invitrogen by Life Technologies). For RT-qPCR of integrin expression, cDNA was then subjected to quantitative real-time PCR reaction using 5 µl of 20 × diluted first strand cDNA reaction, TaqMan Universal PCR Master mix (Appliedbiosystems, Life Technologies LTD, 7 Kingsland Grange, Woolston Warrington, UK) and the following TaqMan Assays (Appliedbiosystems, Life Technologies Corporation, 6055 Sunol Blvd, Pleasanton, CA): Hs00235006_m1 for ITGA1, Hs00233808_m1 for ITGAV, Hs01127536_m1 ITGB1 and Hs00174435_m1 for ITGB5. TBP (TaqMan assay Hs00427620_m1) was used as a reference gene. For HIF1α, ARNT, E2F1 and FOXM1, cDNA was subjected to quantitative real-time PCR using PerfectCT SybR Green Fastmix ROX (Quantabio, Beverly, MA) and the following primers: for FOXM1 forward (fw) 5’-CATTGGACCAGGTGTTTAAG-3’ and reverse (rv) 5’-CCCCTCCTCAGCTAGCAGCAC-3’; for E2F1 fw 5’-GCTGGACCACCTGATGAATATC-3’ and rv 5’- GTCTGCAATGCTACGAAGGT-3’; for HIF-1α fw 5’-CCTAACGTGTTATCTGTCGC-3’ and rv 5’-GTCAGCTGTGGTAATCCACT-3′^88^; for ARNT fw 5’-GGAATGGACTTGGCTCTGTAA-3’ and rv 5’- GTCATCATCTGGGAGGGAAAC-3’. Ribosomal protein Rpl32 was used as a reference gene for SybR Green RT-qPCR and primers were as follows: Rpl32-fw: 5′-CATCTCCTTCTCGGCATCA-3′ and Rpl32-rv: 5′-CTGGGTTTCCGCCAGTTAC-3′^89^. Reactions were run in triplicate. The PCR results were analyzed as relative mRNA level of cycle threshold (CT) value normalized to the scrambled CRISPR/cas9 neuroblastoma cells in each group for each experiment as the control. 

### Migration and invasion assay

Migration and Invasion assays were performed following the Corning Cell Migration and Invasion Quantification Assay with Acetic Acid-dependent Elution of Crystal Violet. Modifications were that SH-SY5Y cells were precultured for 24 h serum free, 2 × 10^5^ cells were added/well, and the migration assay was performed over 48 or 72 h (Fig. 2) and invasion for 3 or 5 days (Fig. 2). For SK-N-AS cells, the migration assay was performed over 24 h, and invasion for 48 h. Corning 24-well plates 8 micron were used for migration and Corning 24-well plates 8 micron coated with Matrigel for invasion assays. In some experiments, cells were incubated with the integrin complex antagonists obtustatin (α1β; R&D Systems, Minneapolis, MN; 0.5, 1 µM), cilengitide (αvβ5; R&D Systems; 1, 5 µM), and GLPG-0187 (αvβ1 and αvβ5; R&D Systems; 5, 10 nM), or the Akt inhibitor afuresertib (Selleckchem, USA; 0.1, 0.5 µM), or ERK inhibitor ravoxertinib (Selleckchem; 0.5, 1 µM). After crystal violet staining and imaging with microscopy and photgraphy, acetic acid elution and quantification were performed with a plate reader (Cell Migration and Invasion Quantification Assay with Acetic Acid-dependent Elution of Crystal Violet, Corning Life Sciences, Tewksbury, Ma).

### Immunoblot analysis

Western blotting was performed as described previously^19^. Cells were grown for 24 h without serum to conform with conditions of cells in migration/invasion assays. Blots were probed with the following antibodies: anti-TRPM2-C (1:1000; Bethyl Laboratories, Montgomery, TX, USA)^90^, anti-V5 (1:2000; Invitrogen, Carlsbad, CA, USA), anti-actin (1:10,000; Sigma, St. Louis, MO, USA), anti-pAkt^ser473^ (1:1000; Cell Signaling Technology INC, Boston, MA, USA), anti-Akt (1:1000; Cell Signaling Technology INC), anti-ARNT (1:1000; Cell Signaling Technology INC), anti-pCREB (no. 9198, 1:250; Cell Signaling Technology INC), anti-CREB (no. 9171, 1:250; Cell Signaling Technology INC), anti-E2F1 (1:1000; Cell Signaling Technology INC), anti-pERK^Thr202/Tyr204^ (1:1000; Cell Signaling Technology INC), anti-ERK (1:1000; Cell Signaling Technology INC), anti-FOXM1 (1:1000; Cell Signaling Technology INC), anti-GAPDH (1:10,000; Cell Signaling Technology INC), anti-HIF-1α (1:500; Cell Signaling Technology INC), anti-integrin α1 (1:1000; Cell Signaling Technology INC), anti-integrin αv (1:1000; Cell Signaling Technology INC), anti-integrin β1 (1:1000; Cell Signaling Technology INC), anti-integrin β5 (1:500; Cell Signaling Technology INC), anti-lamin (1:1000; Cell Signaling Technology INC), anti-Na,K-ATPase α (1:1000, Cell Signaling Technology INC), and anti-tubulin (1:10,000; Sigma). Secondary antibodies were conjugated to IRDye 800CW or IRDye 680RD (donkey anti-rabbit, 1:20,000, or donkey anti-mouse, 1:20,000) and bands quantitated with the Odyssey CLx fluorescence scanner. All bands were analyzed with Image Studio. Samples (Scr, KO) derived from the same experiment were always processed in parallel on a blot. Each blot was probed individually with an antibody, then re-probed with additional single antibodies or two at the same time when molecular weights were sufficiently different (> 30 kDa). The different probes for each antibody were made explicit in the figures with white space and delineated with a black box. For each protein, at least three experiments were performed. For each figure, subgroups shown (A,B, etc.) were from the same experiment unless the high number of antibodies made additional probing not feasible. Blots were cut to maintain 6 band widths above and below the band space permitting.

### Immunoprecipitation

100 µL magnetic beads conjugated to anti-mouse IgG antibodies (Dynabeads M280, Invitrogen, Thermo Fisher Scientific Inc., Waltham, MA, USA), were pre-washed in phosphate buffered saline (PBS) containing protease inhibitors (Thermo Scientific, Rockford, IL, USA) and then incubated with mouse monoclonal integrin α1 (1:50, Invitrogen, Rockford, IL, USA); Integrin αV (1:25, EMD Millipore Corp. USA); Integrin β1 (1:50 Invitrogen, Carlsbad, CA, USA); Integrin β5 (1:25, Invitrogen, Carlsbad, CA); V5 (1:50, Invitrogen, Carlsbad, CA); or mouse serum IgG antibodies (Santa Cruz Biotechnology, Inc. Dallas, TX) overnight on a rocking platform at 4 °C. After a subsequent wash with PBS the beads were incubated with 200 ug total protein on a rocking platform overnight at 4 °C. The beads were then washed five times with PBS containing protease inhibitors, and immunoprecipitated proteins were eluted by adding 50 μl of reducing loading buffer and heating at 70 °C for 10 min. The co-immunoprecipitation was analyzed by Western blotting.

### Subcellular fractionation

Subcellular fractionation was performed using the Subcellular Protein Fractionation Kit (Thermo Scientific, Rockford, IL, USA) according to the manufacturer's instructions. :Whole cell lysates were used to fractionate proteins into membrane, cytoplasmic and nuclear fractions using the manufacturer’s protocol.

### Flow cytometry

Expression of surface integrins was examined using the antibodies: CD49a (integrin alpha1) monoclonal antibody (TS2/7)—PE conjugated (Invitrogen, Carlsbad, CA); CD51 (integrin alpha V) monoclonal antibody (RMV-7)—PE conjugated (Invitrogen, Carlsbad, CA); CD29 (integrin beta1) monoclonal antibody (TS2/16)—PE conjugated (Invitrogen, Carlsbad, CA); and integrin beta 5 monoclonal antibody (KN52)—FITC Conjugated (Invitrogen, Carlsbad, CA). SH-SY5Y KO cells (KO1-V, KO2-V) and KO cells stably expressing TRPM2 (KO1-M2, KO2-M2) (1 × 10^6^ cells) were collected and washed twice in PBS with 2% fetal bovine serum. Non-permeabilized cells were incubated with antibodies listed above on ice for 30 min in the dark. Cells were then washed twice and resuspended in PBS with 2% fetal bovine serum. The cell suspension was immediately analyzed on a flow cytometer.

### Measurement of Ca^2+^

Fluo-4 Direct Kit (Invitrogen, Carlsbad, CA) was used to measure calcium entry in response to hydrogen peroxide stimulation with the Clariostar fluorescent microplate reader (BMG Labtech, Cary, NC). SK-N-AS cells cultured in DMEM with 10% fetal calf serum were incubated with Fluo-4 Direct reagent and then exposed to hydrogen peroxide (0, 500 µM). Fluorescence was read every 2 min for 30 min. Data was analyzed by two-way ANOVA with Tukey’s procedure for multiple comparisons.

### Statistical analysis

Results are expressed as mean + S.E.M. unless otherwise noted. For analysis of most experiments, one-way ANOVA, or T-test was used as noted in Figure Legends and *p* < 0.05 was taken to be statistically significant. Two-way ANOVA was used where specified.

### Ethics statement

This study does not require ethical approval.

## Supplementary Information

Supplementary Information.

## Data Availability

The data generated during and/or analyzed in the current study are available from the corresponding author on reasonable request. RNA seq data have been deposited in NCBI’s Gene Expression Omnibus and are accessible through GEO Series accession number GSE203660.
