# Supplementary material for: The human ion channel TRPM2 modulates migration and invasion in neuroblastoma through regulation of integrin expression
Source: Sci Rep. 2022 Nov 29;12:20544. doi: 10.1038/s41598-022-25138-w (PMC9709080; doi:10.1038/s41598-022-25138-w)
Supplement: Supplementary file 1 — Supplementary Information. [file 41598_2022_25138_MOESM1_ESM.pdf]

Supplemental Information File

The human ion channel TRPM2 modulates migration and invasion in neuroblastoma through regulation of integrin expression

Lei Bao[1], Fernanda Festa[1,2], Iwona Hirschler-Laszkiewicz[1], Kerry Keefer[1], Hong-Gang Wang[1,3], Joseph Y. Cheung[4], and Barbara A. Miller[1,2*]

Affiliations: Departments of [1]Pediatrics, [2]Biochemistry and Molecular Biology, and [3]Pharmacology, The Pennsylvania State University College of Medicine, P.O. Box 850, Hershey, Pa 17033, USA

[4]Renal Medicine, Brigham and Women's Hospital, Boston, MA 02115, USA

Supplementary Figure S1

Supplementary Figure S1. Full length gels for Western blot images used to create Figure 2 A are shown. Western blots were probed with antibodies to detect V5-labeled TRPM2 (left) and actin (right) as a loading control. Blots were cut around the expected molecular weights for each protein after probing. Red rectangles mark the bands used.

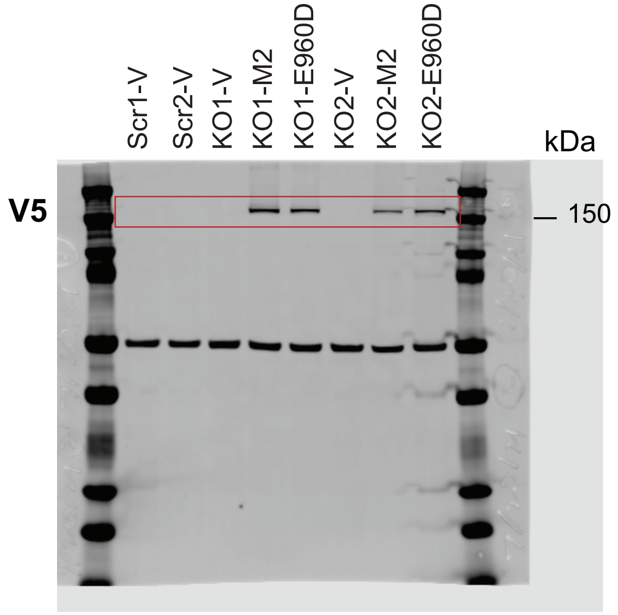

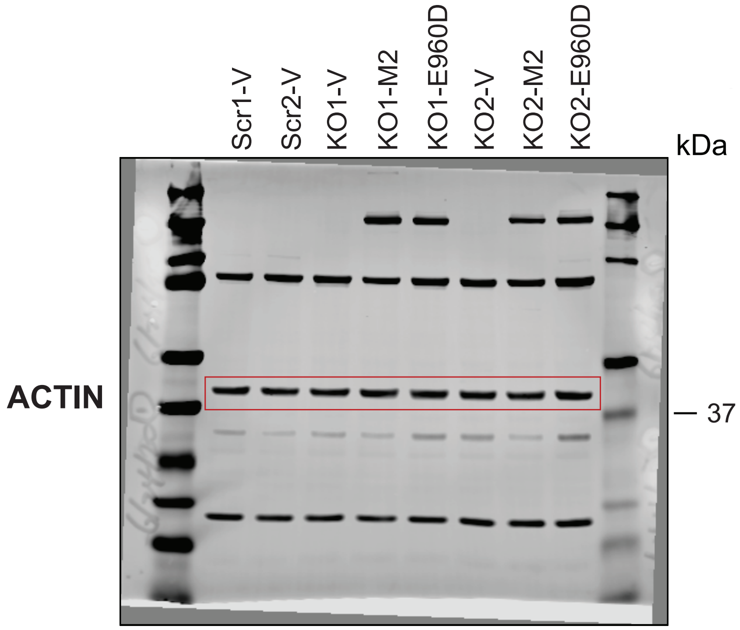

Supplementary Figure S2

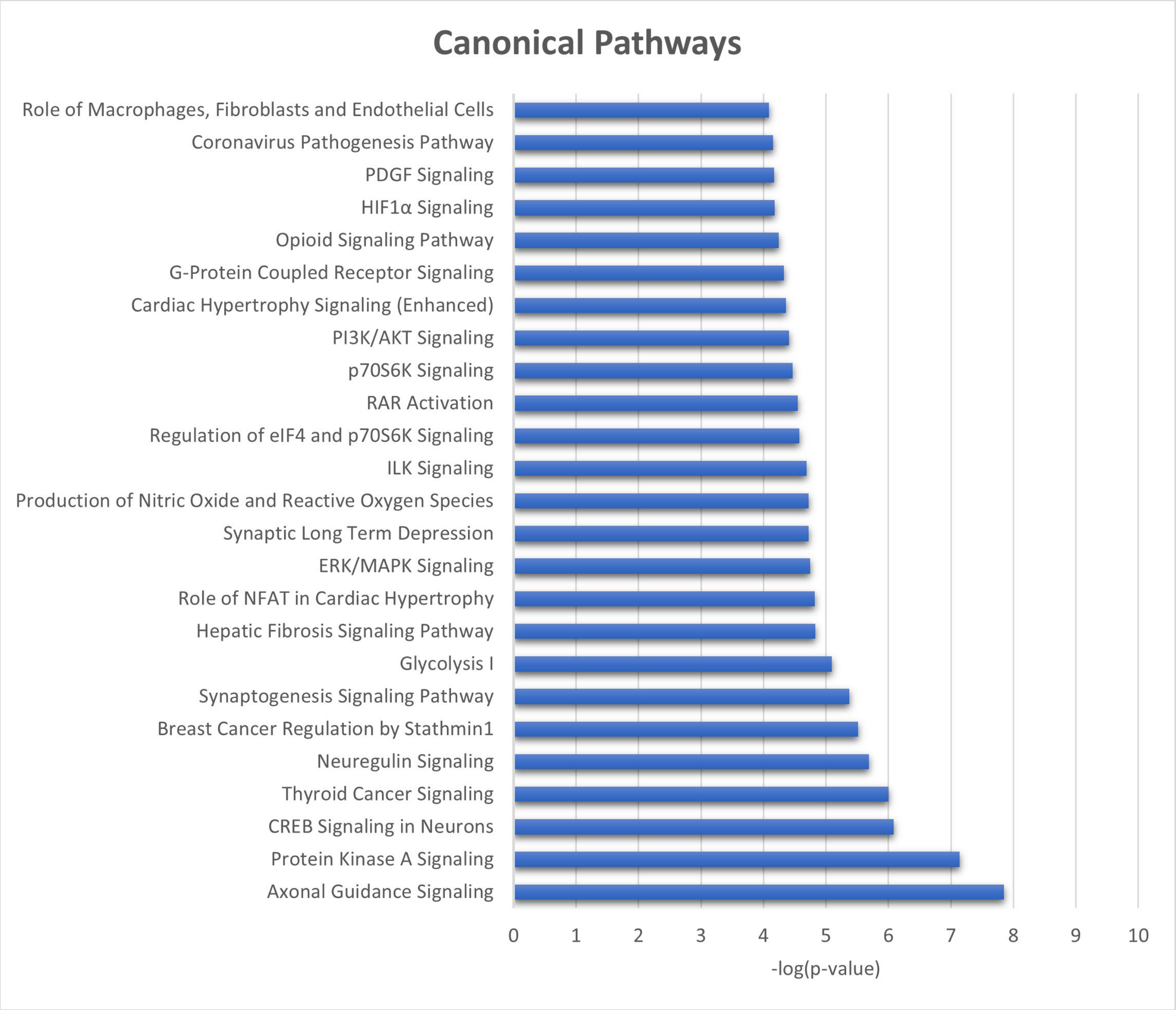

Supplementary Figure S2. Top 25 canonical pathways modulated by high TRPM2 expression in neuroblastoma identified by RNA seq. RNA seq was performed using neuroblastoma SH-SY5Y cells with TRPM2 deletion (SH-SY5Y KO) with or without reconstitution with TRPM2 (SH-SY5Y TRPM2). One clone for each cell line was tested in duplicate. Genes with q-value <0.05 were considered differentially expressed and the pathway analysis was performed by IPA. The list of the top 25 pathways is displayed in order of increasing statistical significance.

Supplementary Figure S3

Supplementary Figure S3B. RT-qPCR of integrin ITGA1, ITGAV, ITGB1, ITGB5 mRNA from Scr-V (average of Scr1-V and Scr2-V), KO2-V, KO2-M2, or KO2-E960D transfected SH-SY5Y cells. Each experimental group was normalized to combined Scr clones. Means ± S.E.M. of three (ARNT), four (ITGA1, ITGAV, ITGB1), or seven (ITGB5) experiments are shown. Statistics: one-way ANOVA, *p<0.05, **p<0.01, ***p<0.001, ****p<0.0001.

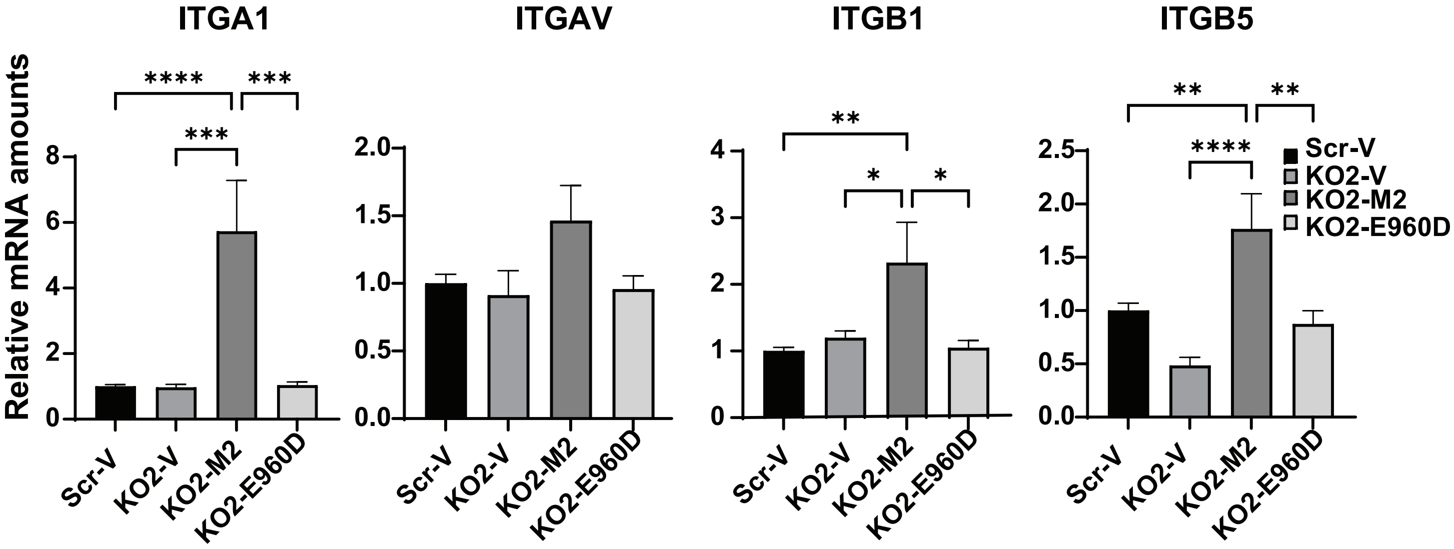

Supplementary Figure S3 continued

Supplementary Figure S3C. Full length gels for Western blot images used to create Figure 3C are shown. Western blots were probed with antibodies to Integrins (ITGA1, ITGAV, ITGB1, ITGB5). Tubulin was used as a loading control. Blots were cut around the expected molecular weights for each protein after probing. Red rectangles mark the bands used.

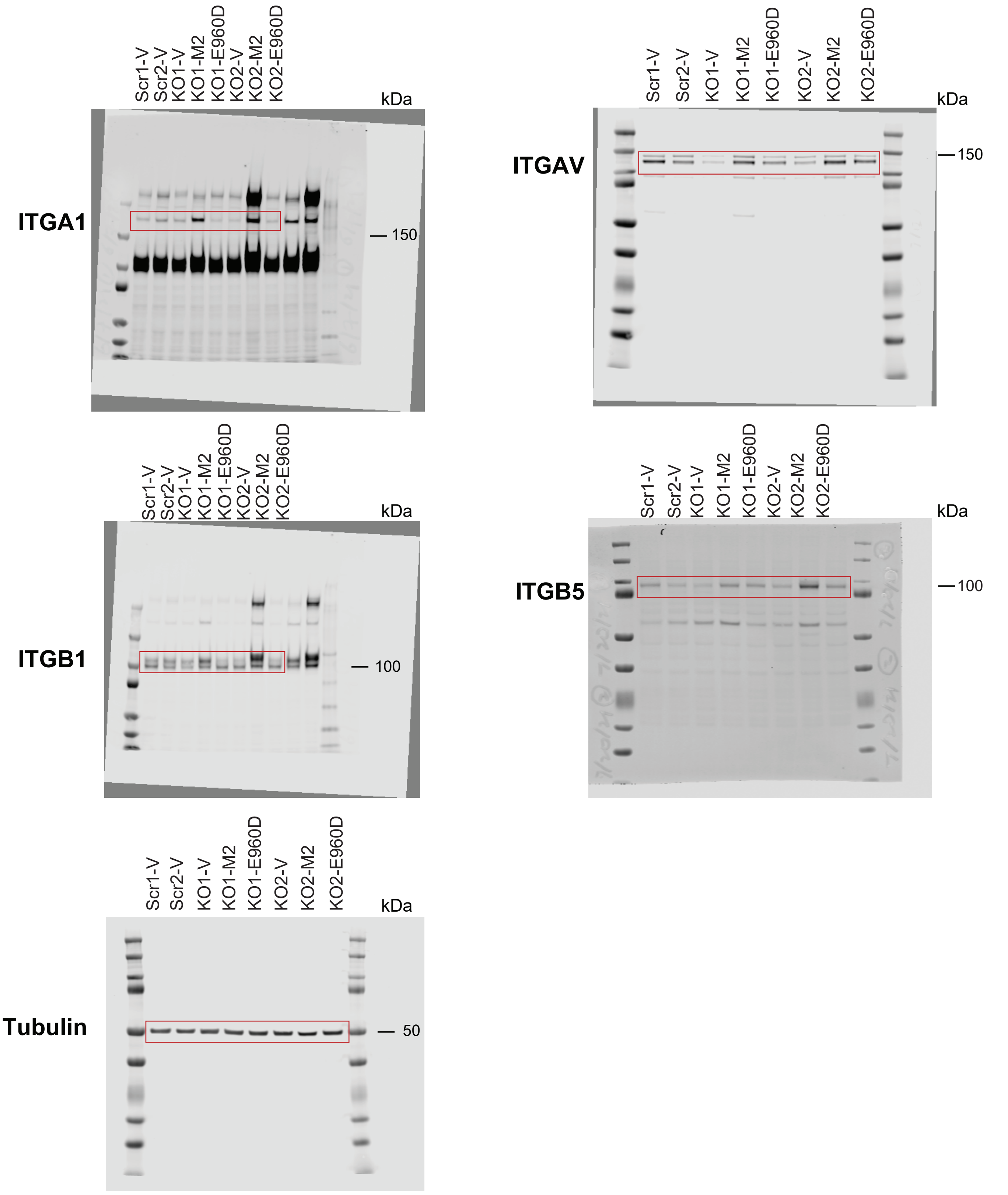

Supplementary Figure S3 continued

Supplementary Figure S3C. Western blotting was performed on a second clone of TRPM2 KO cells (KO2-V), KO reconstituted with TRPM2 (KO2-M2) or E960D (KO2-E960D), and scrambled SH-SY5Y control cells (Scr1-V, Scr2-V). Blots were probed with antibodies to α1 (ITGA1), αv (ITGAV), β1 (ITGB1), β5 (ITGB5) integrins and tubulin as a loading control. Densitometry measurements from five experiments for each integrin (n=5) standardized to each experiment's scrambled control were analyzed. Means ± S.E.M. are shown. Statistics: one-way ANOVA, *p<0.05, **p<0.01, ***p<0.001, ****p<0.0001.

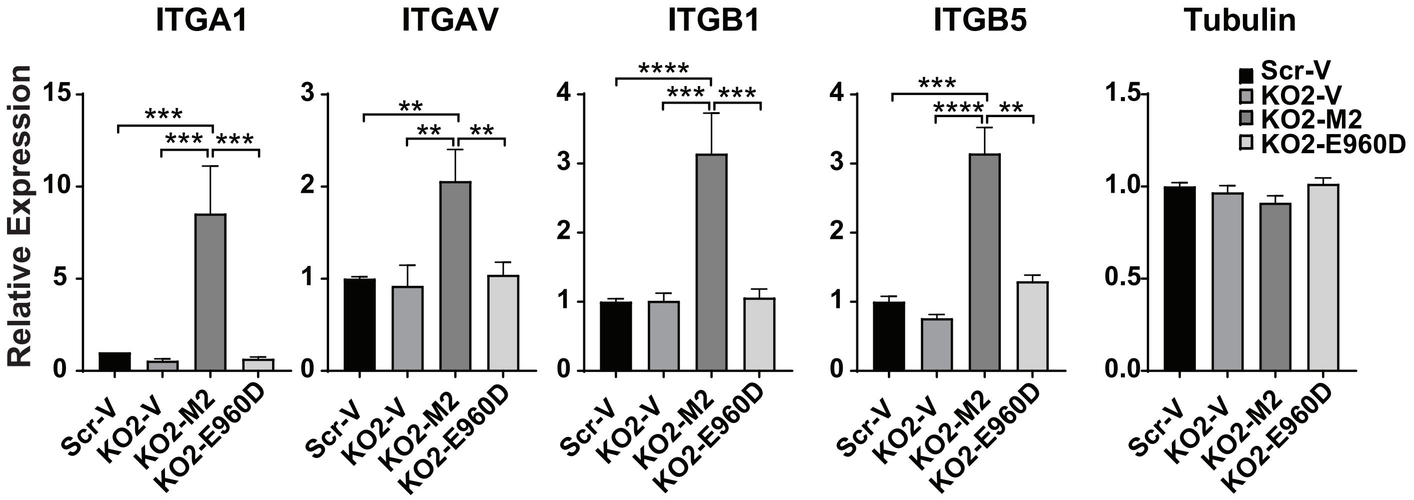

Supplementary Figure S3 continued

Supplementary Figure S3D. RT-qPCR of transcription factors HIF-1α, E2F1, FOXM1, and ARNT mRNA from Scr-V (average of Scr1-V and Scr2-V), KO2-V, KO2-M2 or KO2-E960D expressing SH-SY5Y cells. Each experimental group was normalized to Scr. Means ± S.E.M. of three (ARNT), four (HIF1A), or five (E2F1, FOXM1) experiments are shown. Statistics: one-way ANOVA, *p<0.05, **p<0.01, ***p<0.001, ****p<0.0001.

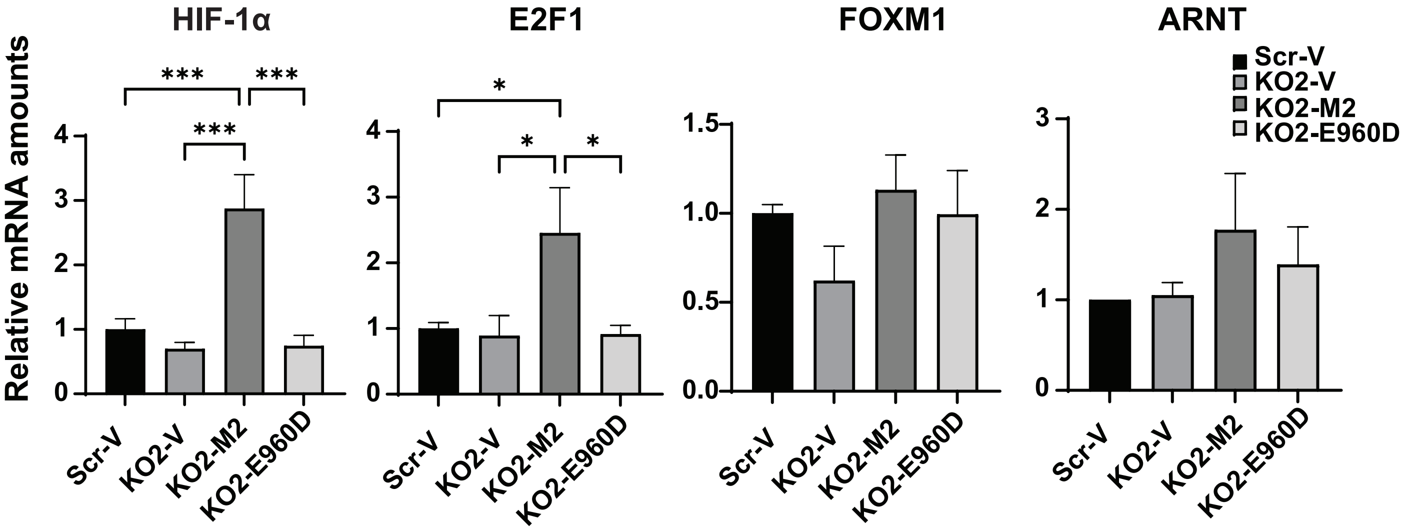

Supplementary Figure S3 continued

Supplementary Figure S3E. Full length gels for Western blot images used to create Figure 3 E are shown. Western blots were probed with antibodies to transcription factors HIF-1α, E2F1, FOXM1, and ARNT. Tubulin was used as a loading control. Blots were cut around the expected molecular weights for each protein after probing. Red rectangles mark the bands used.

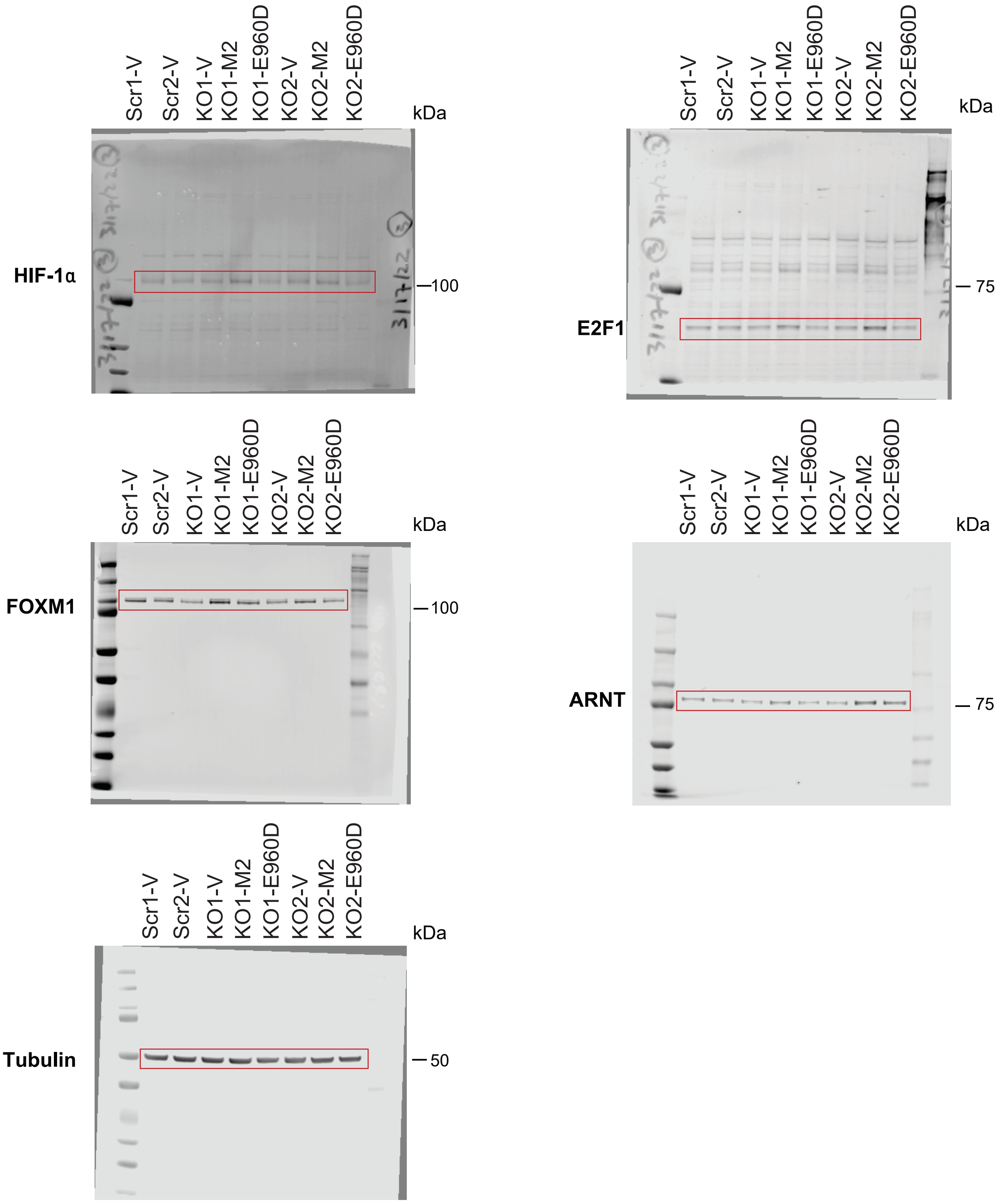

Supplementary Figure S3 continued

Supplementary Figure S3E. Western blotting was performed on a second clone of TRPM2 KO (KO2-V), KO reconstituted with TRPM2 (KO2-M2) or E960D (KO2-E960D), and scrambled SH-SY5Y control cells (Scr1-V, Scr2-V). Blots were probed with antibodies to transcription factors HIF-1α, E2F1, FOXM1, ARNT, and tubulin as a loading control. Densitometry measurements from five experiments for transcription factors HIF-1α, E2F1, and FOXM1 (n=5), and eight experiments for ARNT (n=8) were standardized to each experiment's scrambled control. Means ± S.E.M. for each group are shown. Statistics: one-way ANOVA, *p<0.05, **p<0.01, ***p<0.001, ****p<0.0001.

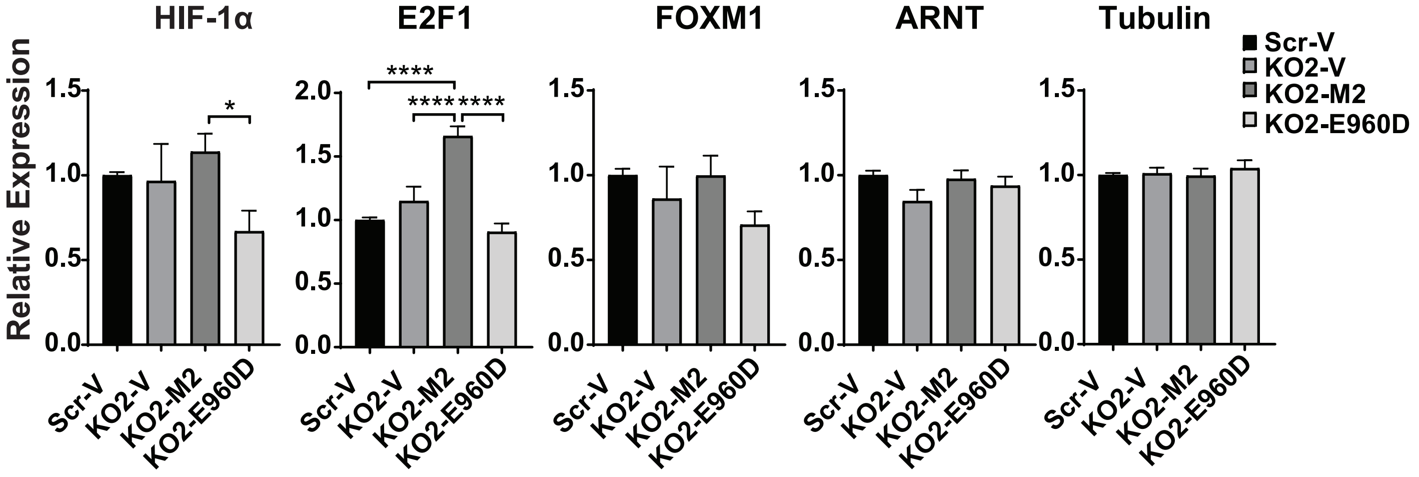

Supplementary Figure S4

Supplementary Figure S4. Full length gels for Western blot images used to create Figure 4 are shown. Following subcellular fractionation of two KO clones transfected to express TRPM2, Western blotting was performed and blots were probed with antibodies to ITGA1, ITGAV, ITGB1, and ITGB5. Blots were also probed with antibodies to GAPDH, Na,K-ATPase, and Lamin A/C as controls for quality of fractionation. Blots were cut around the expected molecular weights for each protein after probing. Red rectangles mark the bands used.

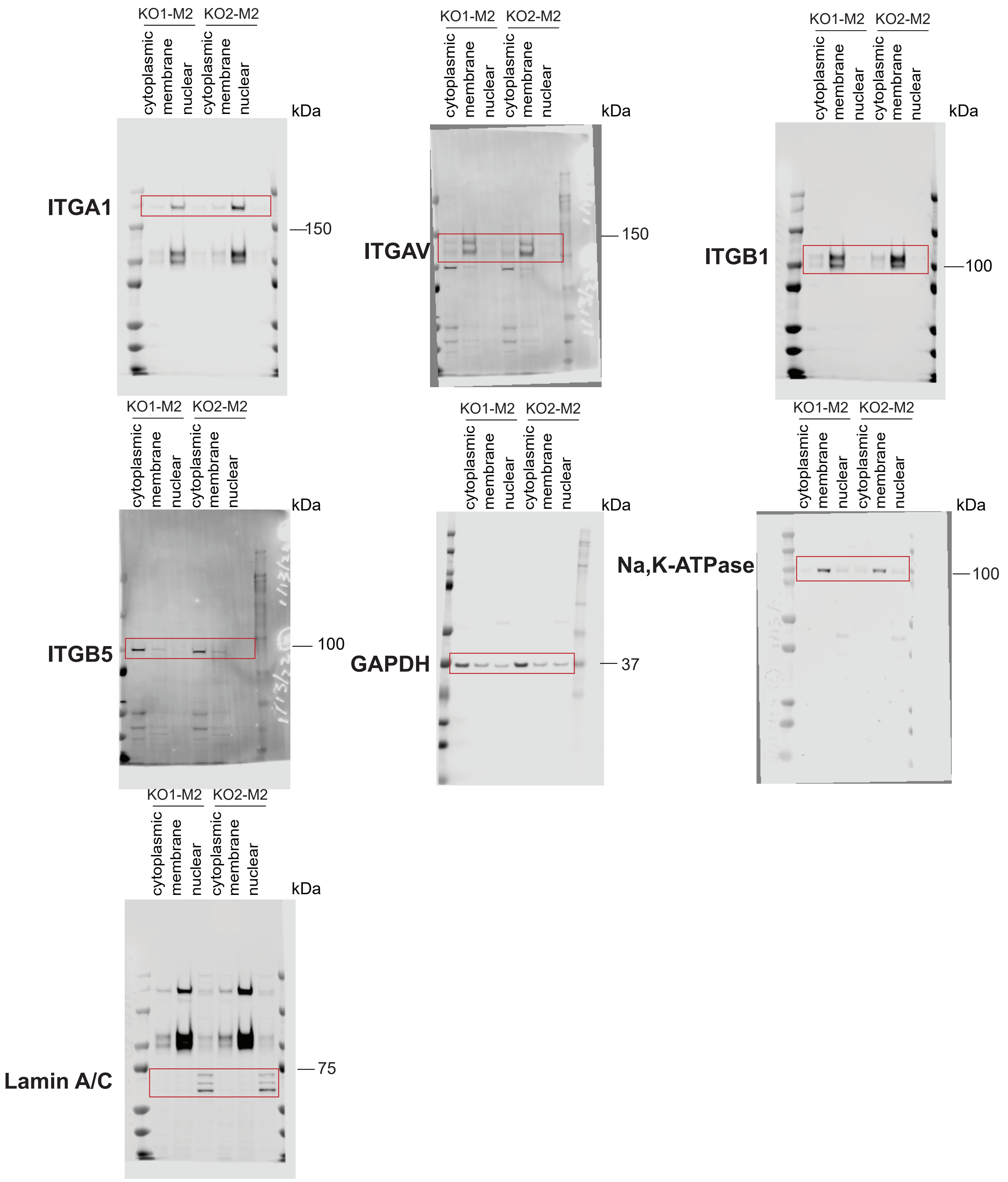

Supplementary Figure S4 continued

Supplementary Figure S4.Subcellular Fractionation of α1, αv, β1, and β5 integrins in neuroblastoma cells highly expressing TRPM2. Subcellular separation of two clones of SH-SY5Y scrambled control cells (Scr1-V, Scr2-V), KO cells (KO1-V, KO2-V), KO cells reconstituted with TRPM2 (KO1-M2, KO2-M2) or E960D (KO1-E960D, KO2-E960D) into membrane fractions was performed. Western blotting was performed with fractionated samples loading equivalent amounts per lane (10 ug/lane). Blots were probed with antibodies to α1 (ITGA1), αv (ITGAV), β1 (ITGB1), β5 (ITGB5) integrins. Blots were also probed with antibodies to GAPDH, Na,K-ATPase α and Lamin A/C as controls. Results of one experiment of two are shown.

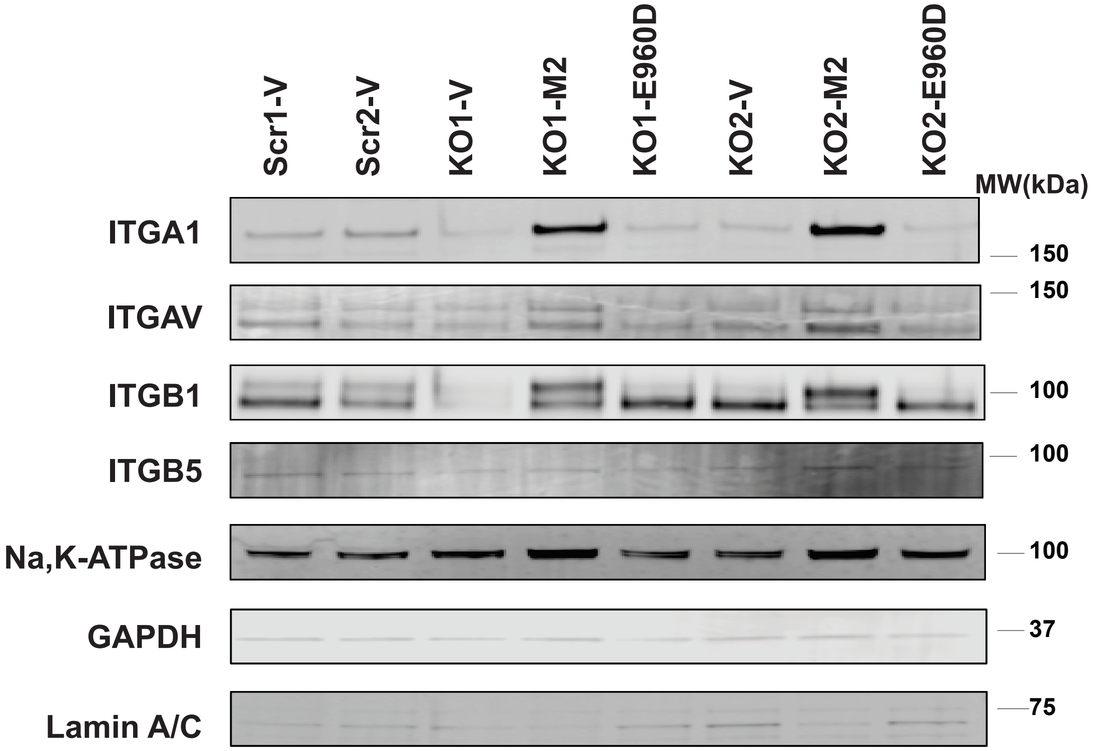

Supplementary Figure S5

Supplementary Figure S5. Full length gels for Western blot images used to create Figure 5 are shown. Following immunoprecipitation with the antibody shown at the top of each column, Western blotting was performed with non-binding supernatants and immunoprecipitates. Blots were probed with antibodies to integrins ITGA1, ITGB1, ITGAV, and ITGB5 and to TRPM2. Blots were cut around the expected molecular weights for each protein after probing. Red rectangles mark the bands used.

Full Length Blots Figure 5

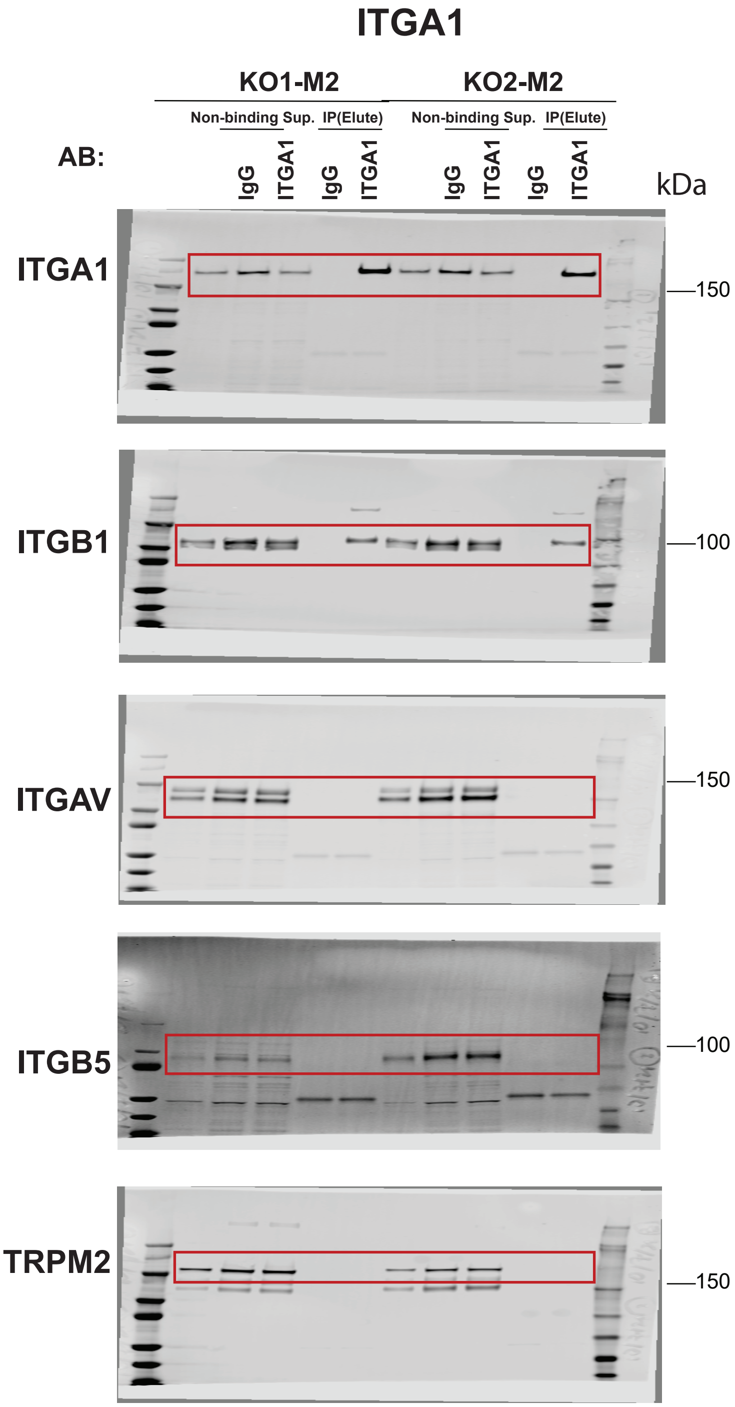

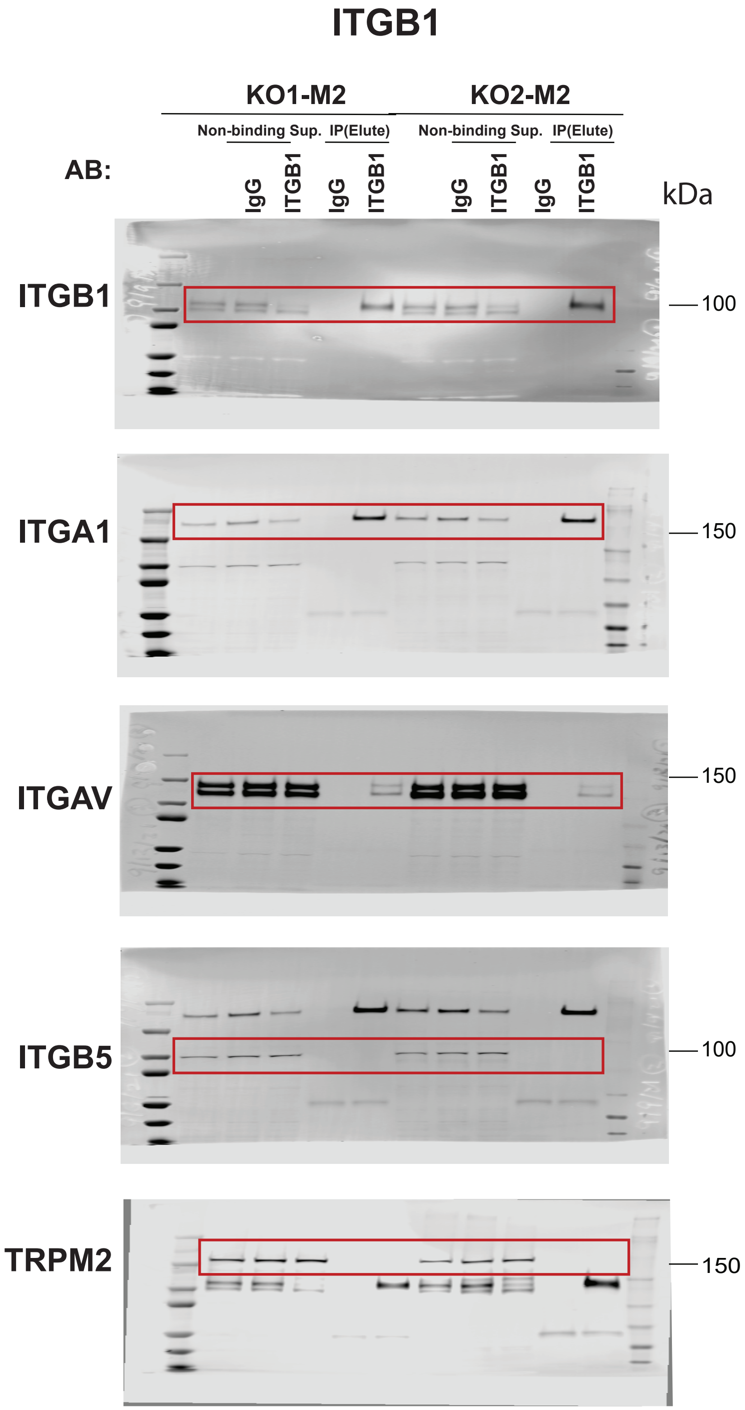

Supplementary Figure S5 continued

Full Length Blots Figure 5

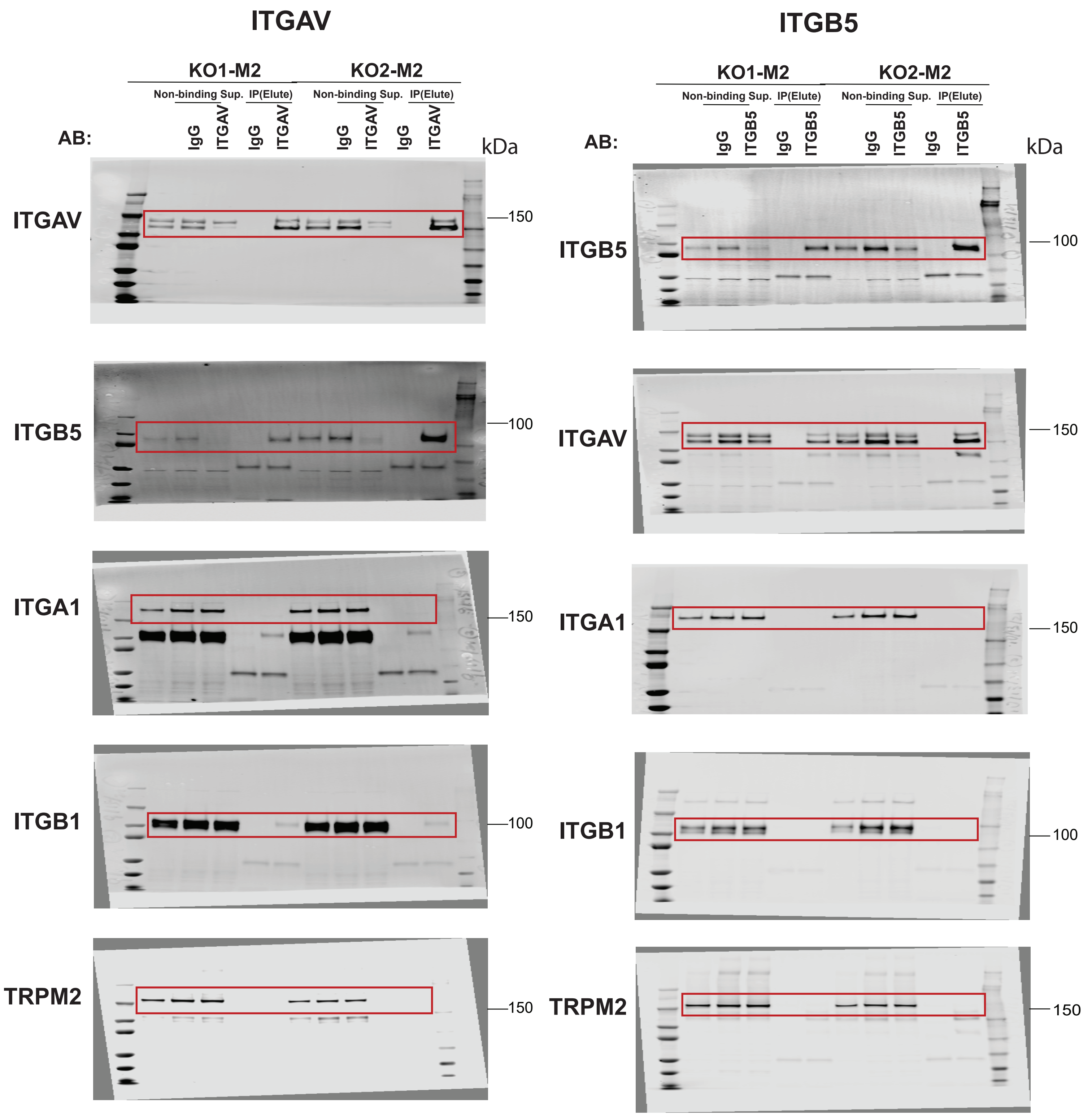

Supplementary Figure S5 continued

Full Length Blots Figure 5

TRPM2

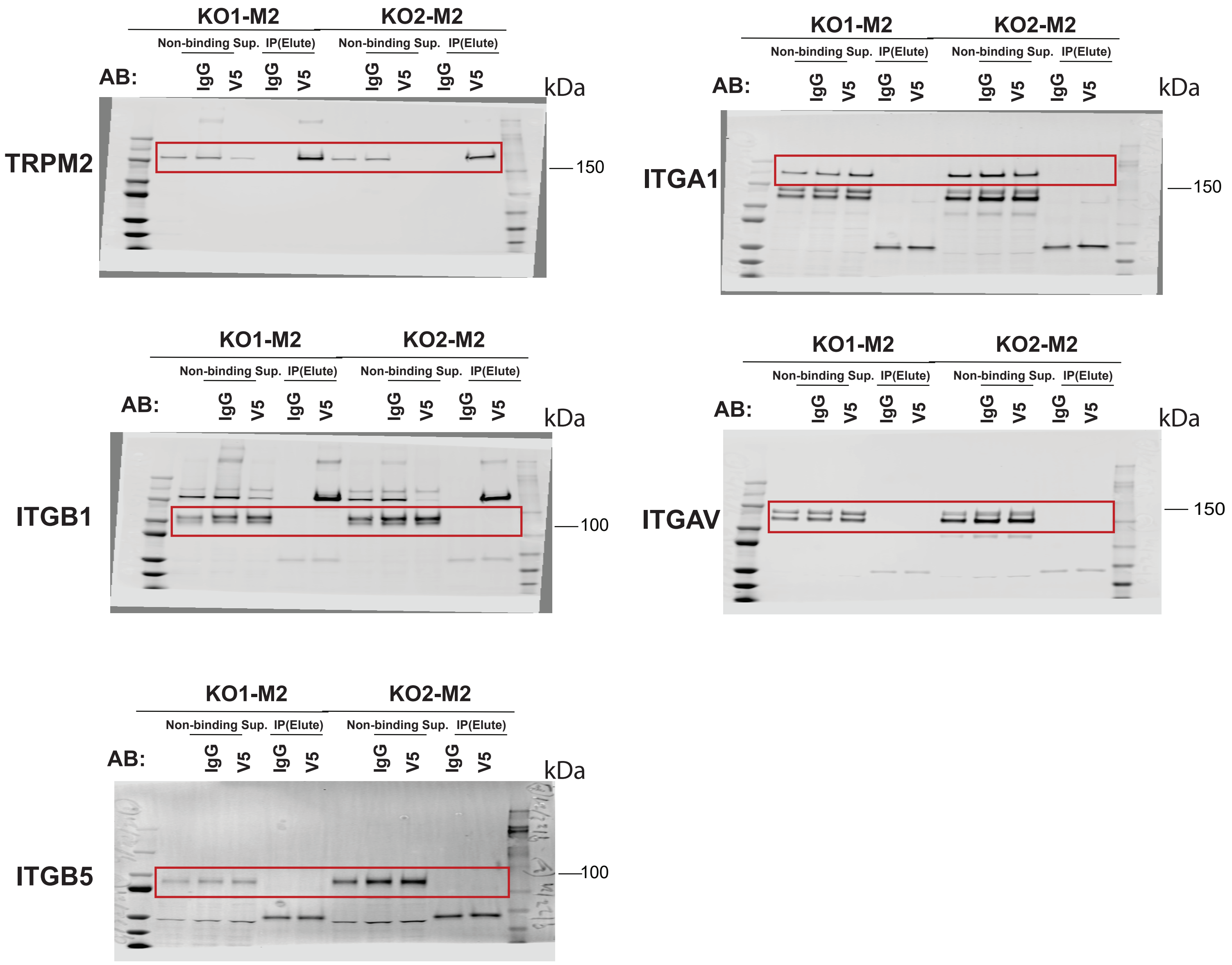

Supplementary Figure S6

Supplementary Figure S6. Cell viability following treatment with integrin complex or Akt or ERK inhibitors. Viability was examined with XTT analysis following treatment of two clones each of SH-SY5Y TRPM2 knockout cells (KO1-V, KO2-V) or KO cells reconstituted to express TRPM2 (KO1-M2, KO2-M2) with integrin complex (**A**) or Akt or ERK inhibitors (**C**). In (**B**), Scr (Scr1-V, Scr2-V) and KO1,2-E960D cells were also treated with integrin inhibitors.

A: Integrin Complex Inhibitors: KO-V (KO1-V, KO2-V) or KO-M2 (KO1-M2, KO2-M2) cells were treated with integrin complex inhibitors obtustatin (0, 0.5, 1 µM), cilengitide (0, 1 µM, 5 µM) or GLPG-0187 (0, 5, 10 nM) for 24, 48, or 72 hours. Results (OD 490-690) were normalized to untreated cells at each time point for each clone (n=4). Means ± S.E.M. are shown and results analyzed with two-way ANOVA. *p<0.05, indicates significantly less than untreated cells.

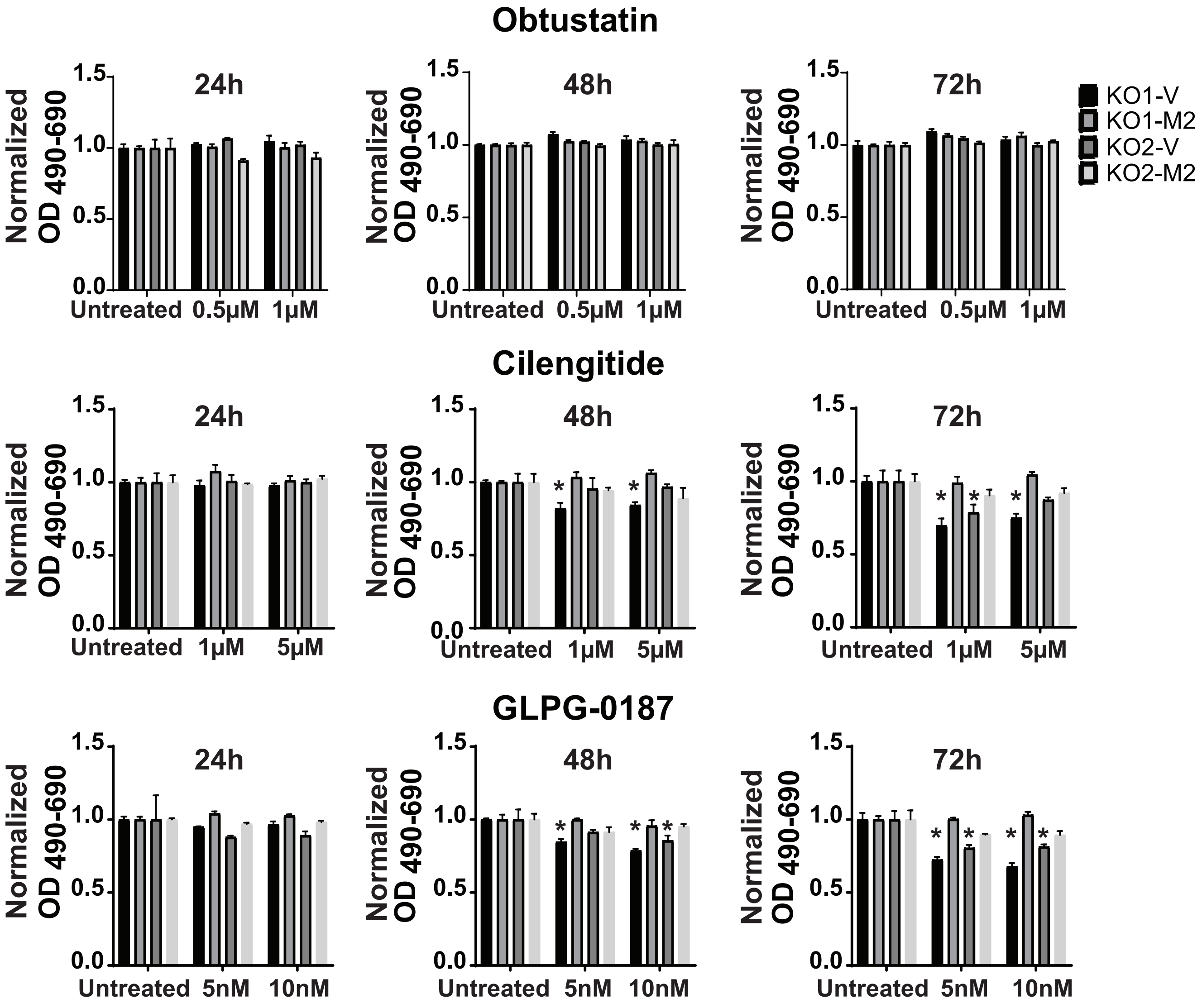

Supplementary Figure S6 continued

B: Scr-V (Scr1-V, Scr2-V), KO-V (KO1-V, KO2-V), and KO clones stably expressing TRPM2 (KO1-M2, KO2-M2) or E960D (KO1-E960D, KO2-E960D) were also treated with obtustatin (1 μM), cilengitide (1, 5 μM), and GLPG-0187 (10, 20 nM) for 24 or 48 hours (n=3/clone/group). Results (OD 490-690) were normalized to untreated cells at each time point for each clone (n=3). Means ± S.E.M. are shown and results analyzed with two-way ANOVA. *p<0.05, **p<0.01, indicates significantly less than untreated cells.

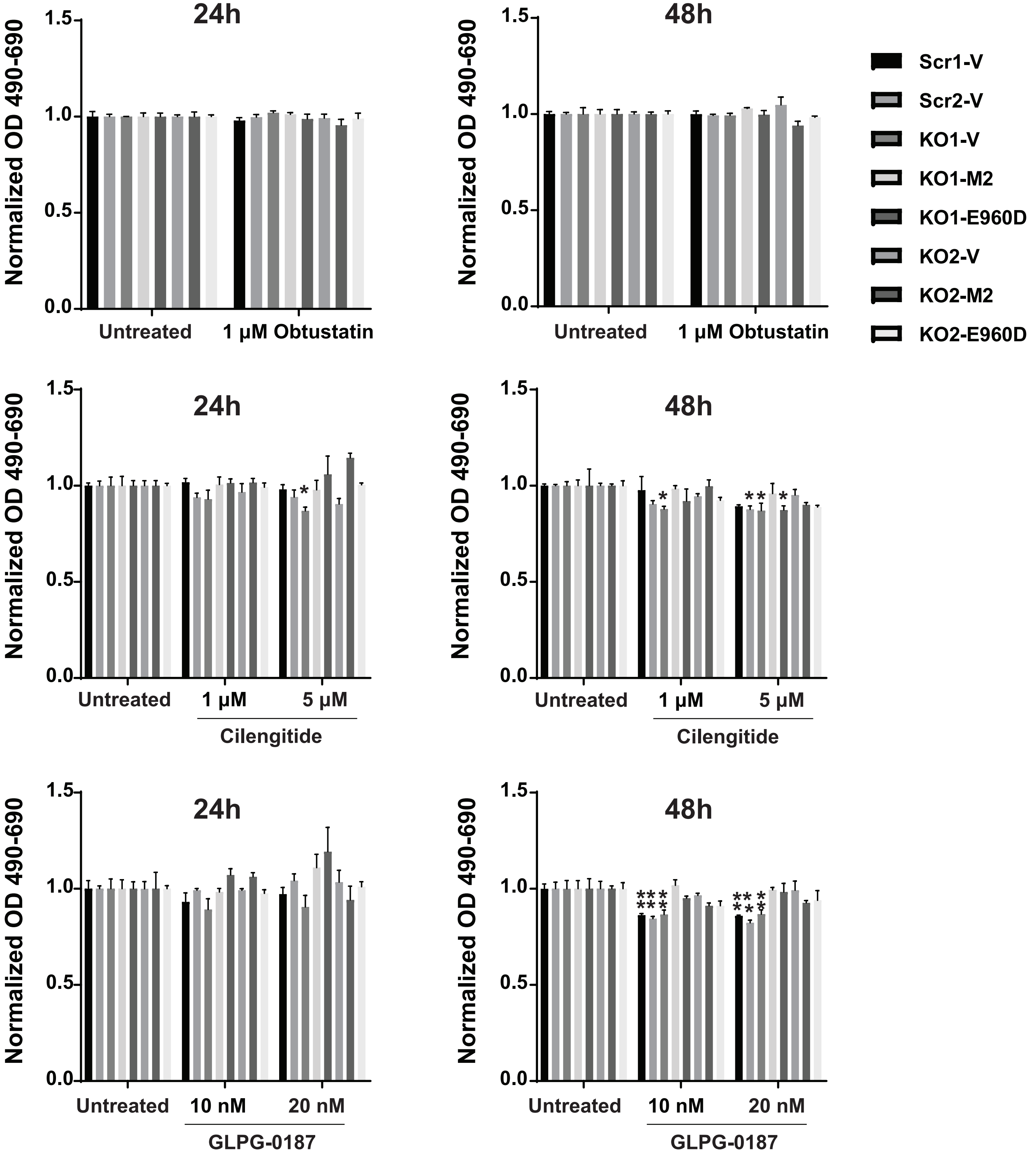

Supplementary Figure S6 continued

C: Akt and ERK Inhibitors: KO-V (KO1-V, KO2-V) or KO-M2 (KO1-M2, KO2-M2) cells were treated with the Akt inhibitor afuresertib (0, 0.1, 0.5 μM) or the ERK inhibitor ravoxertinib (0, 0.5, 1 μM) for 24, 48, or 72 hours. Results (OD 490-690) were normalized to untreated cells at each time point for each clone (n=4). Means ± S.E.M. are shown and results analyzed with two-way ANOVA. *p<0.05, indicates significantly less than untreated cells.

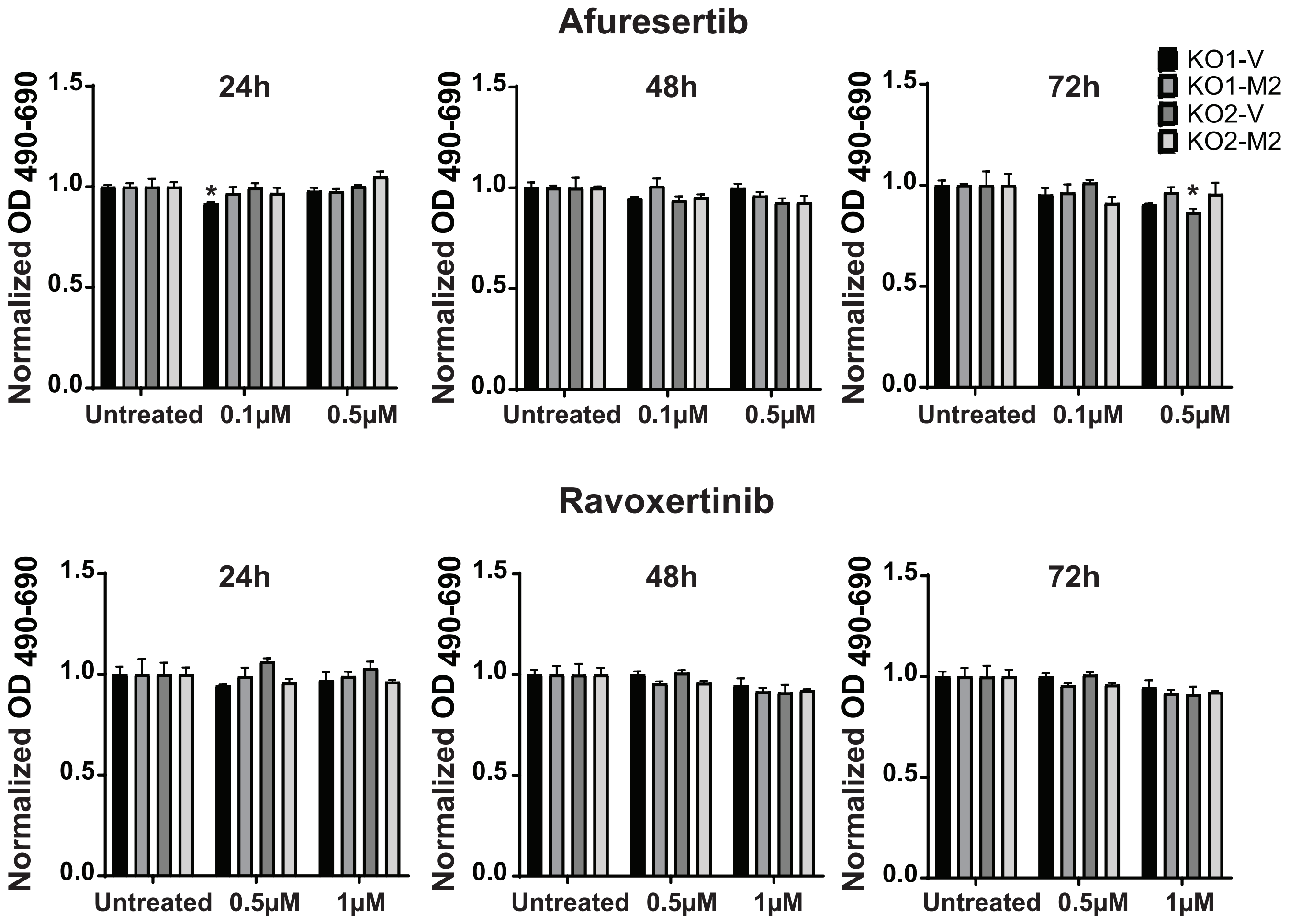

Supplementary Figure S7

Supplementary Figure S7. Full length gels for Western blot images used to create Figure 7 A are shown. Western blots were probed with antibodies to phosphorylated Akt (p-Akt-Ser473), Akt, phosphorylated ERK (p-ERK-Thr202/Tyr204), and ERK. Tubulin was used as a loading control. Blots were cut around the expected molecular weights for each protein after probing. Red rectangles mark the bands used.

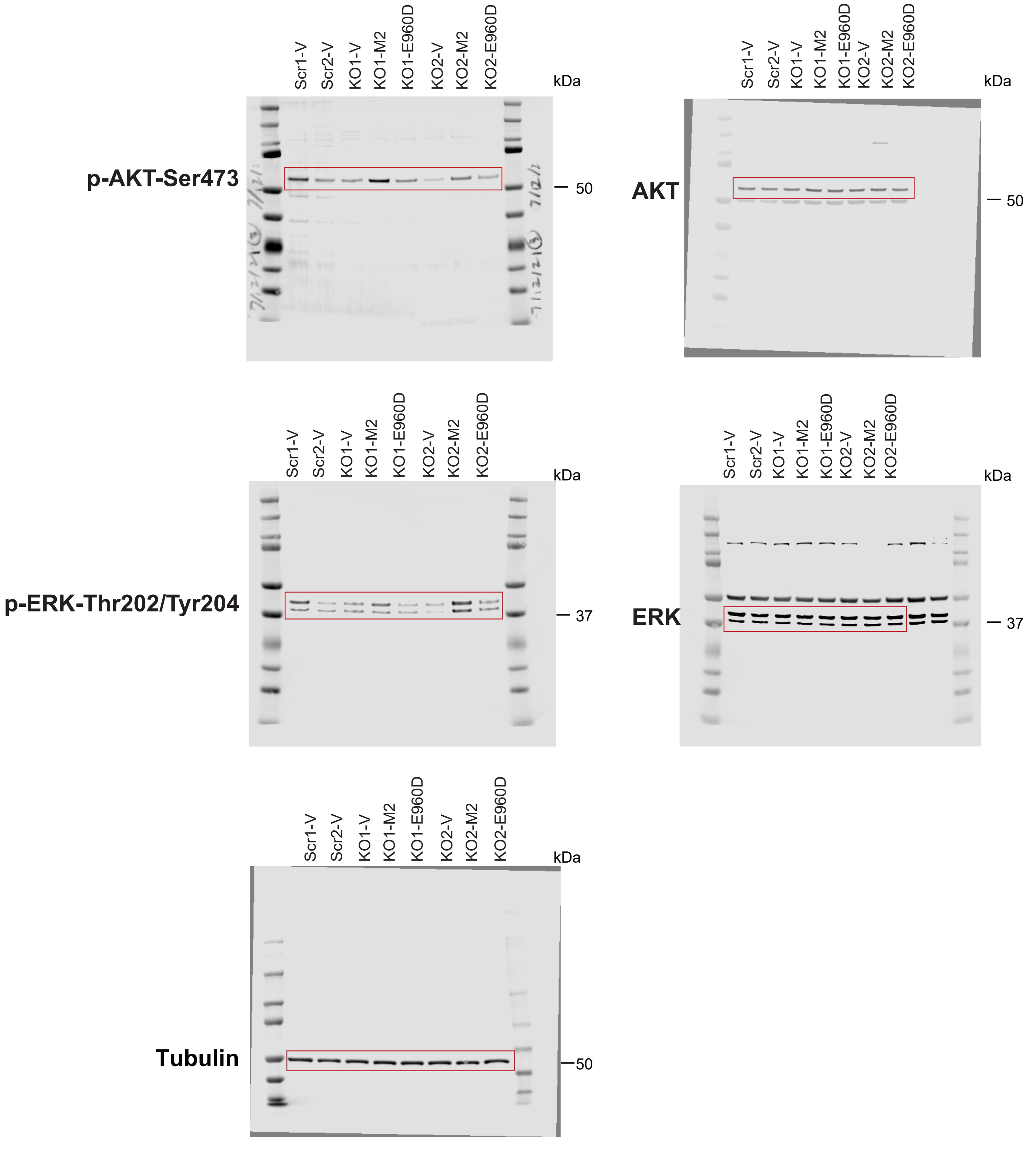

Supplementary Figure S7 continued

Supplementary Figure S7A. Western blots of lysates from SH-SY5Y KO2 cells (KO2-V), KO2 cells stably expressing TRPM2 (KO2-M2), E960D (KO2-E960D), or scrambled control cells (Scr1-V, Scr2-V) were probed with antibodies to pAktSer473, Akt, pERKThr202/Tyr204, ERK, and tubulin in four experiments. Densitometry measurements were standardized to scrambled control for each blot. Means ± S.E.M. are shown for the four experiments (n=4). Statistics: one-way ANOVA, *p<0.05, **p<0.01, ***p<0.001.

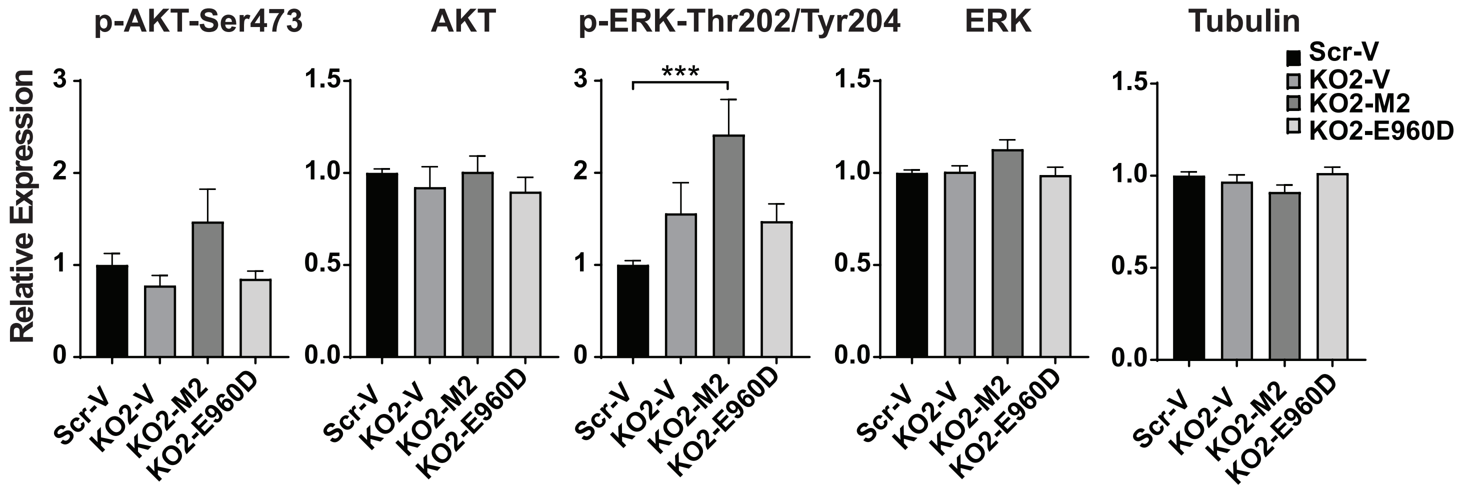

Supplementary Figure S8

Supplementary Figure S8 A. RT-qPCR of TRPM2 mRNA from SK-N-AS wild type (WT), scrambled control (Scr), and TRPM2 KO cells. Primers recognized sequences in TRPM2 exons 2-3, exons 3-4, or the TRPM2 c-terminal end. RT-qPCR demonstrated depletion of TRPM2 with primers recognizing exons 3-4 and the 3' end in SK-N-AS TRPM2 KO cells. Each RT-PCR was run in triplicate.

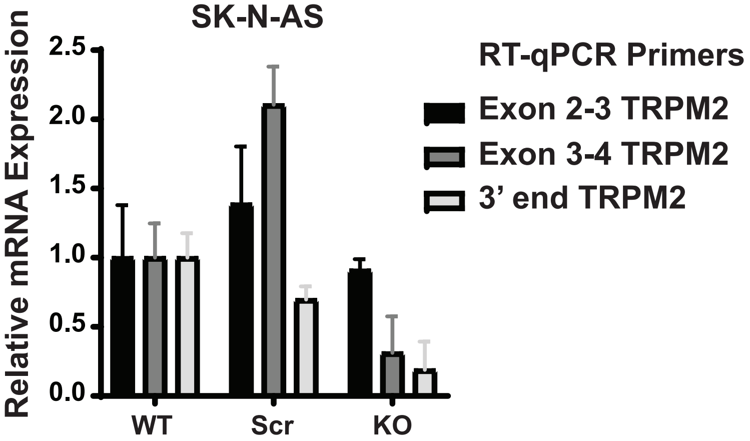

Supplementary Figure S8 B. Calcium entry in Fluo-4 loaded SK-N-AS wild type, scrambled, and knockout cells. Fluorescence intensity was measured at 2 minute intervals for 30 minutes after treatment with 0 or 500 μM hydrogen peroxide with the Clariostar. Three similar experiments were performed and results of one are shown. Means ± S.E.M. fluorescence intensity at each time point are shown (n=16 replicates/time point). Statistics: two-way ANOVA, *p≤0.05, **p≤0.0001 Wt and Scr 500 μM H2O2 vs KO 500 μM H2O2.

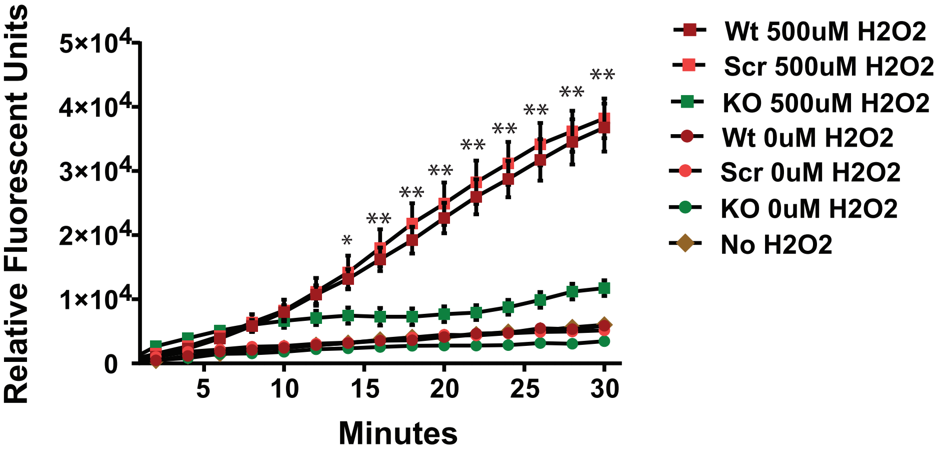

Supplementary Figure S8 C. Migration and invasion assays were performed using SK-N-AS KO cells (KO) and scrambled control (Scr). Pictures of two additional migration and invasion experiments used in statistical analysis in Figure 8 B are shown.

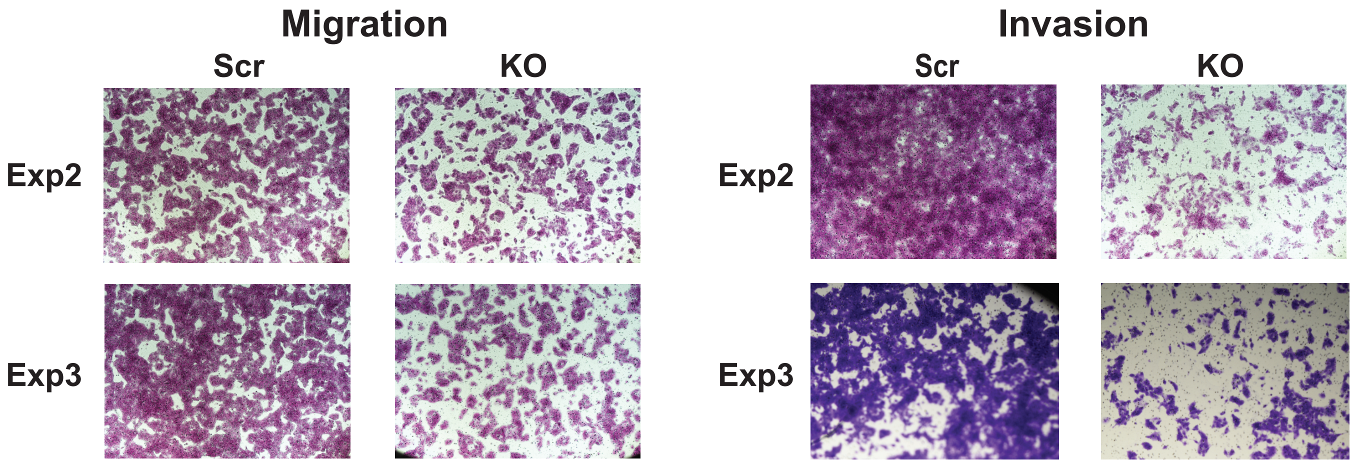

Supplementary Table 1

TCGA Abbreviation	Cancer Type	Number of Samples	
		Normal	Tumor
BLCA	Bladder Urothelial Carcinoma	28	404
BRCA	Breast invasive carcinoma	291	1085
CESC	Cervical squamous cell carcinoma and endocervical adenocarcinoma	13	306
COAD	Colon adenocarcinoma	349	275
ESCA	Esophageal carcinoma	286	182
HNSC	Head and Neck squamous cell carcinoma	44	519
KIRC	Kidney renal clear cell carcinoma	100	523
KIRP	Kidney renal papillary cell carcinoma	60	286
LAML	Acute Myeloid Leukemia	70	173
OV	Ovarian serous cystadenocarcinoma	88	426
PAAD	Pancreatic adenocarcinoma	171	179
READ	Rectum adenocarcinoma	318	92
SKCM	Skin Cutaneous Melanoma	558	461
STAD	Stomach adenocarcinoma	211	408
UCEC	Uterine Corpus Endometrial Carcinoma	91	174
UCS	Uterine Carcinosarcoma	78	57
TOTAL		2756	5550

Supplementary Table 1 – Cancer types with high TRPM2 expression. GEPIA2 tool was used to measure the levels of TRPM2 in 32 cancer types against normal control samples and 16 tumors with statistically different expression of TRPM2 are reported. The TCGA abbreviation, cancer type and the number of normal and tumor samples for each group are displayed.

Supplementary Table 2

	Number of sample (MYCN-amp per stage %)												
	Stage1		Stage2		Stage3		Stage4		Stage4S				
**Database**	non-MYCN	MYCN-amp	non-MYCN	MYCN-amp	non-MYCN	MYCN-amp	non-MYCN	MYCN-amp	non-MYCN	MYCN-amp	TOTAL	survival	PMID
**Cangelosi**	137	5 (3.5%)	120	5 (4%)	84	21 (20%)	198	116 (36.5%)	85	6 (6.6%)	786	Yes	32825087
**Kocak**	114	5 (4.2%)	75	4 (5.1%)	55	15 (21.4%)	105	65 (38.2%)	56	4 (6.7%)	649	Yes	23579273
**Westermann**	305*	28* (8.4%)*	305*	28* (8.4%)*	305*	28*(8.4%)*	151	78 (34.1%)	305*	28*(8.4%)*	579	No	NA
**Seeger**							102				102	Yes	16954472

* Combined data for stage 1, 2, 3 and 4S
NA - not applicable

Supplementary Table 2 – Neuroblastoma datasets used for TRPM2 expression and survival expression profiles. All datasets were obtained from the R2 platform.
